# Signal amplification by cyclic extension enables high-sensitivity single-cell mass cytometry

**DOI:** 10.1038/s41587-024-02316-x

**Published:** 2024-07-29

**Authors:** Xiao-Kang Lun, Kuanwei Sheng, Xueyang Yu, Ching Yeung Lam, Gokul Gowri, Matthew Serrata, Yunhao Zhai, Hanquan Su, Jingyi Luan, Youngeun Kim, Donald E. Ingber, Hartland W. Jackson, Michael B. Yaffe, Peng Yin

**Affiliations:** 1 Wyss Institute for Biologically Inspired Engineering, Harvard University, Boston, MA USA; 2 Departments of Biology and Bioengineering, Koch Institute for Integrative Cancer Research, MIT Center for Precision Cancer Medicine, Massachusetts Institute of Technology, Cambridge, MA USA; 3 Mount Sinai Health Systems and Department of Molecular Genetics, Lunenfeld Tanenbaum Research Institute, University of Toronto, Toronto, Ontario Canada; 4 Department of Systems Biology, Harvard Medical School, Boston, MA USA; 5 Harvard John A. Paulson School of Engineering and Applied Sciences, Harvard University, Cambridge, MA USA; 6 Vascular Biology Program and Department of Surgery, Harvard Medical School and Boston Children’s Hospital, Boston, MA USA; 7 Department of Surgery, Beth Israel Deaconess Medical Center, Divisions of Acute Care Surgery, Trauma, and Critical Care and Surgical Oncology, Harvard Medical School, Boston, MA USA; 8 Present Address: Department of Materials Science and Engineering, Seoul National University, Seoul, Republic of Korea

**Keywords:** Proteomics, DNA, Signal processing

## Abstract

Mass cytometry uses metal-isotope-tagged antibodies to label targets of interest, which enables simultaneous measurements of ~50 proteins or protein modifications in millions of single cells, but its sensitivity is limited. Here, we present a signal amplification technology, termed Amplification by Cyclic Extension (ACE), implementing thermal-cycling-based DNA in situ concatenation in combination with 3-cyanovinylcarbazole phosphoramidite-based DNA crosslinking to enable signal amplification simultaneously on >30 protein epitopes. We demonstrate the utility of ACE in low-abundance protein quantification with suspension mass cytometry to characterize molecular reprogramming during the epithelial-to-mesenchymal transition as well as the mesenchymal-to-epithelial transition. We show the capability of ACE to quantify the dynamics of signaling network responses in human T lymphocytes. We further present the application of ACE in imaging mass cytometry-based multiparametric tissue imaging to identify tissue compartments and profile spatial aspects related to pathological states in polycystic kidney tissues.

## Abstract (web summary)

Mass cytometry with signal amplification enables measurement of low-abundance proteins.

## Main

Mass cytometry allows quantitative measurement of up to 50 proteins or protein modifications at single-cell resolution^1^, enabling the profiling of complex cellular behaviors in highly heterogeneous samples^2–6^. Coupled to a high-resolution laser ablation system, imaging mass cytometry (IMC) quantifies targets of interest in antibody-stained adherent cells or tissue sections, with preserved subcellular level (1 μm) spatial information^7,8^. However, current mass cytometry analyses have limited sensitivity, typically requiring the binding of hundreds of metal-tagged antibodies to each species of cellular epitope to reach the detection threshold of the instrument^9,10^. This has prevented the development of mass cytometry for analysis of components of the low-abundance proteome, including many transcription factors, surface receptor proteins and intracellular phosphorylation sites, which play important roles in health and disease^9–11^.

In conventional mass cytometry, metal isotopes are chelated onto maleimide-modified diethylenetriamine pentaacetate (DTPA) polymers, which are conjugated directly to the reactive cysteine residues of a partially reduced antibody^1^. Each antibody carries a limited number of metal ions. One approach to amplify signal in mass cytometry is by increasing the metal-antibody stoichiometry, but this has been challenging to date. Existing signal amplification methods face technical difficulties in their implementation for mass cytometry. For example, both tyramide signal amplification (TSA)^12^ and alkaline phosphatase-mediated amplification^13^ apply enzyme-conjugated antibodies to catalyze signal accumulation around target proteins, but such methods introduce high nonspecific signals and do not allow multiplexing. Rolling circle amplification (RCA) uses circular DNA to rapidly synthesize hybridization sites of detection oligonucleotide strands^14^. By designing orthogonal DNA sequences in the circular DNA, RCA has achieved high multiplexing in single-cell RNA quantification^15^. However, when combined with antibody-based detection, RCA reactions tend to suffer from nonspecific background antibody binding, which generates high-intensity false-positive signals^16^. In addition, molecular crowdedness often affects the amplification efficiency in an RCA reaction^17^. In hybridization chain reaction (HCR), fluorescently labeled DNA monomers can be induced to assemble on a target DNA primer conjugated to an antibody^18^. The multiplexing limitation of HCR, however, makes it difficult to measure more than a handful of protein epitopes simultaneously^19^. We previously developed the signal amplification by exchange reaction (SABER)^20^ method, which uses presynthesized DNA concatemers to generate signal amplification. Using orthogonal amplifier sequences, Immuno-SABER^21^ can measure tens of protein epitopes sequentially. Recently, this method has been adapted for IMC-based spatial tissue analysis^22^. Nevertheless, applying Immuno-SABER to suspension mass cytometry to amplify the metal ion signal has been difficult because the stringent washing conditions required to remove nonspecific concatemer bindings in imaging samples cannot be applied to cells in suspension. Additionally, DNA duplexes are unstable during the high-temperature single-cell droplet vaporization step before the time-of-flight mass analysis^1^, compromising the amplification power of Immuno-SABER.

Here, to address the sensitivity limitation in current mass cytometry approaches, we create a signal amplification approach in which DNA barcodes conjugated on an antibody undergo repeated in situ extension under thermal-cycling conditions, generating multiple copies of detector oligonucleotide binding sites. By using nine-mer short DNA barcodes as initiators labeled on antibodies, we reduce nonspecific binding often caused by long DNA oligonucleotides used as antibody barcodes as seen in other methods^19,21,23^. This thermal-cycling-based in situ extension chemistry has demonstrated successful results in amplifying signals for fluorescence microscopy imaging (unpublished data). In mass cytometry analysis, we additionally incorporate a 3-cyanovinylcarbazole phosphoramidite (CNVK)-based nucleic acid photocrosslinking method^24,25^, which allows our amplification DNA structures to achieve the high thermal stability required for mass cytometry sample introduction. We show that our method, termed Amplification by Cyclic Extension (ACE), enables over 500-fold signal amplifications with uncompromised signal-to-noise ratios. We validated 33 orthogonal ACE sequences with an average of 1.07% of channel-to-channel crosstalk. Applying a 32-parameter ACE panel to profile molecular signatures during the epithelial-to-mesenchymal transition (EMT) and the less studied mesenchymal-to-epithelial transition (MET) in mouse Py2T cells^26^, our analysis identified that the expression ratio between Zeb1 and cyclin B1 can function as a hallmark for cells to undergo MET. Extending ACE to a second application, we simultaneously amplified 30 T cell receptor (TCR) signaling markers to comprehensively profile the TCR signaling network in human Jurkat T-cells and primary human CD4^+^ T cells during a 1-h stimulation timecourse. In depth analysis of TCR network dynamics in response to patient postoperative drainage fluid (POF) costimulation revealed an immunosuppressive T cell signature caused by tissue injury. Coupling ACE with IMC-based tissue imaging to profile the structural organization and phenotypical heterogeneity in human kidney tissues, our analyses recapitulated six main compartments in renal cortexes and revealed the heterogeneous expression levels of a stemness marker—nestin—in polycystic kidney disease. In summary, ACE provides an approach to address sensitivity challenges in mass cytometry analysis, enabling the profiling of low-abundance proteomic substrates in single cells.

## Results

### Signal amplification through thermal cyclic primer extension

**Fig. 1:**
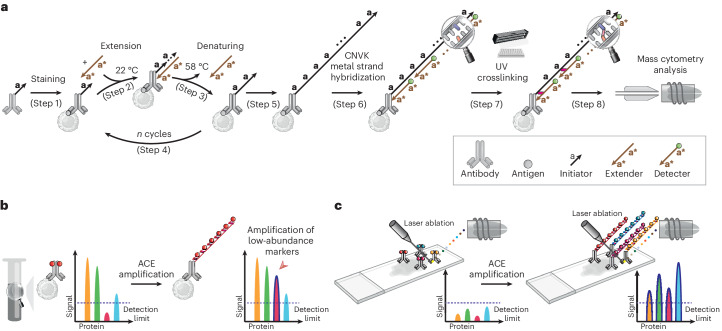
Schematic of ACE and its applications. **a**, Schematic of the ACE approach for signal amplification in mass cytometry analysis. **b**, ACE amplifies the signal in suspension mass cytometry, allowing detection and quantification of low-abundance markers that do not reach detection limits of a mass cytometer using conventional approaches. **c**, Coupled with IMC, ACE enables high-sensitivity multiparametric spatial profiling of healthy and diseased tissue samples.

To increase the sensitivity of mass cytometry analysis, synthetic oligonucleotides were used to create repeats of metal probe hybridization sites using thermal cyclic extension, amplifying the number of metal ions carried by an antibody (Fig. 1a). Antibodies targeting the protein of interest were first conjugated to short DNA oligonucleotide initiators (TT-**a**, in total 11-mer, **a** = 9-mer). Mixtures of conjugated antibodies were then applied to cell suspensions for cell surface or intracellular marker staining (step 1). Next, an extender oligonucleotide containing two repeats of sequence complementary to the initiator, separated by a deoxythymidine spacer (a*-T-a*, 19-mer) was introduced to the stained cells. At 22 °C, the extender and initiator hybridize, allowing *Bst* polymerase-mediated initiator strand extension (forming TT-**a**-A-**a**-A, step 2, *Bst* polymerase adds a single A base to the 3′ end after each round of primer extension). Following this, the reaction temperature was raised to 58 °C, to denature the initiator–extender hybrid, and expose the extended initiator strand (TT-**a**-A-**a**-A, step 3). These thermal cycles (1 min per cycle) were then repeated to successively elongate the initiator (step 4), creating hundreds of **a**-A repeats on each antibody conjugation site (step 5). Detectors containing the sequence a*-T-a* were then conjugated to DTPA polymers containing chelated Ln^3+^ metal ions through a maleimide-thiol reaction. Hundreds of metal-conjugated detectors were hybridized to each extended initiator (step 6), thereby substantially amplifying the metal signal on a per antibody basis.

Initial experiments applying ACE for mass cytometry resulted in low signals and high acquisition backgrounds (Supplementary Fig. 1a, top right panel), suggesting that metal signals were leaking from measured cells. In experiments using fluorescently labeled detectors that could be analyzed by conventional flow cytometry, loss of signal from the detector was reproduced by heating cells for 1 min at 55 °C after ACE, resulting in a greater than 90% fluorescent signal decrease compared with the unheated samples (Supplementary Fig. 1b). In mass cytometry analysis, heat-mediated vaporization of single-cell droplets during the acquisition step denatures DNA double helices and detaches hybridized metal-conjugated detectors, compromising the power of amplification by ACE. To address the above issue, we introduced a photocrosslinking method that incorporates a CNVK^24,25^ modification in the metal-conjugated detector oligonucleotide. Following the detector hybridization step, a brief (1 s) exposure to ultraviolet (UV) light activates the CNVK photocrosslinker, forming a covalent bond between the detector and a deoxythymidine nucleotide on the complementary hybridized DNA strand (Fig. 1, step 7, and Supplementary Fig 1a, middle panel). This stabilized the ACE amplificaiton complex against heat-induced double-helix denaturing, allowing it to remain intact during 55 °C incubation as assessed by flow cytometry (Supplementary Fig. 1b), and during mass cytometry vaporization (Fig. 1, step 8, and Supplementary Fig. 1a, bottom right panel). The ACE approach can be used on protein epitopes of interest in suspension mass cytometry to facilitate low-abundance marker quantification in single-cell samples (Fig. 1b), or can be applied to imaging mass cytometry to enable highly sensitive spatial analysis of tissue specimens (Fig. 1c). Compared with conventional mass cytometry workflows, the ACE protocol (Supplementary Fig. 2a) requires a total additional cost of ~US$24 for a 30-target amplification experiment (Supplementary Fig. 2b).

### ACE enables high-sensitivity mass cytometry analysis

**Fig. 2:**
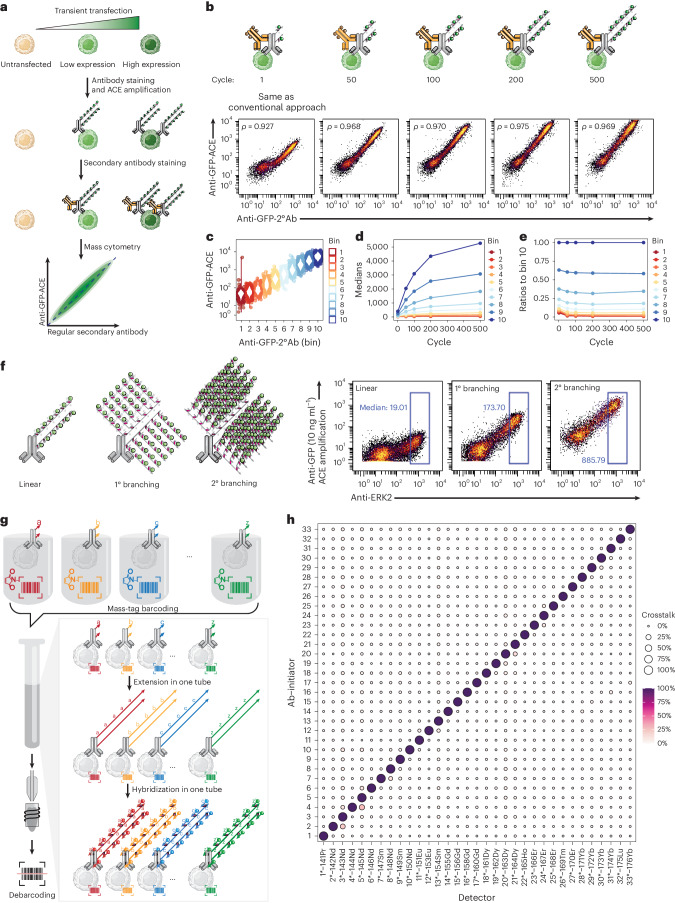
Validation and quantification of ACE for mass cytometry signal amplification. **a**, HEK293T cells transiently transfected with a GFP-encoding plasmid were used to validate the ACE amplification method. **b**, Signal amplification by ACE (*y* axis) through 1–500 thermal cycles was compared with counterstaining using a secondary antibody conjugated with the conventional protocol (*x* axis) to validate the specificity and quantify the amplification efficiency of ACE. Pearson correlation coefficients between ACE and the regular secondary antibody signals were calculated for each condition as a measure of ACE specificity. **c**, Data were divided into ten equal-width bins according to their GFP expression levels shown by the secondary antibody. Bins 1–3 reflect the untransfected cells as internal controls. Bin 10 shows the cells with the highest GFP expression levels. **d**, Bin medians across 1–500 thermal cycles. **e**, Ratio of each bin median over the median in bin 10 across 1–500 thermal cycles. **f**, GFP ion counts generated with ACE after one or two rounds of branching were compared with that from a linear amplification without branching in a ERK2–GFP transient expression experiment using ultralow amount of the GFP antibody (10 ng ml^−1^). A conventionally conjugated anti-ERK2 antibody was used as counterstaining. **g**, To examine the orthogonality of ACE, GFP-expressing HEK293T cells were stained individually with anti-GFP ACE antibodies conjugated to 33 initiator sequences before they were barcoded, pooled and processed through the ACE protocol in the same tube, and then analyzed on a mass cytometer. **h**, Data were then debarcoded to allow pairwise analysis of potential ACE crosstalk. Ion counts generated by a detector in all conditions (columns) were normalized to 0–1 before the ratio between any unmatched initiator–detector pair (for example, Ab-initiator 1–Detector 2*-^142^Nd) and the true signal (for example, Ab-initiator 1–Detector 1*-^141^Pr) was calculated. On average, ACE has 1.02% of crosstalk signal. Four pairs of probes were detected with crosstalk degrees over 10% (Initiator 2–Detector 3*, Initiator 4–Detector 5*, Initiator 7–Detector 8*, Initiator 20–Detector 21*). 1°, primary; 2°, secondary; Ab, antibody.

To explore the specificity and amplification power of ACE, we applied ACE to human embryonic kidney HEK293T cells transiently transfected with a plasmid encoding the green fluorescent protein (GFP). Transient transfection yields a high GFP expression gradient, which allows assessment of ACE to quantify target proteins with different abundance (Fig. 2a). A side-by-side comparison between ACE amplification, conventional fluorescently labeled antibodies and immuno-SABER amplification on flow cytometry revealed that the signal-to-noise ratio in ACE was 3.6-fold higher than that obtained by secondary antibody amplification, and 27-fold higher in comparison with immuno-SABER amplifcation^21^ (Supplementary Fig. 3).

Next, GFP-expressing cells were stained with oligonucleotide-conjugated rat anti-GFP antibodies for ACE amplification through 1–500 thermal cycles, followed by ^172^Yb-labeled detector hybridization. Conventionally labeled ^159^Tb anti-rat secondary antibodies^27^ were used to detect the anti-GFP antibody in the same cells, which were later analyzed by mass cytometry (Fig. 2b). In the absence of cyclic amplification (that is, after the first thermal cycle), the ACE signal of the primary anti-GFP antibody is correlated strongly with the secondary anti-rat antibody, which was metal-conjugated using the conventional protocol (Pearson correlation coefficient = 0.927; Fig. 2b), validating the specificity of the ACE for intracellular epitope staining. Repeated thermal cycles resulted in a continual increase in the ACE ^172^Yb signal from GFP-positive cells relative to the ^159^Tb-labeled secondary antibody, while the signal from GFP negative cells remained at the same levels. Notably, this led to increased Pearson correlations (to a maximum of 0.975) between ACE and the regular secondary antibody (Fig. 2b). We then discretized the data according to the secondary antibody signal (*x* axis) into ten equal-width bins (Fig. 2c and Supplementary Fig. 4a), and calculated the bin medians over thermal cycles. This revealed that ACE amplification was most efficient in the first 100 cycles (about 2 h), after which the amplification efficiency declined over time (Fig. 2d). A 13-fold amplification strength and a sixfold enhancement of signal-to-noise ratio were observed in samples with 500-cycle amplification, compared with the unamplified control (Supplementary Fig. 4b). Untransfected cells (GFP negative, bins 1–3) were not amplified through the timeseries, confirming the specificity of our approach (Supplementary Fig. 4b). The consistency of signal ratios in relation to the highest signal (median levels in bin 10) was maintained throughout the 500-cycle amplification process, indicating that ACE amplification does not introduce bias into the linearity of mass cytometry signals (Fig. 2e). We further validated that the thermal cycles did not affect signals of prestained conventional mass cytometry antibodies (Supplementary Fig. 4c,d). After cyclic extension, DNA concatemers were shown to be stable at 4 °C, which suggests high reproducibility despite varied sample storage time (Supplementary Fig. 4e). We also demonstrated that ACE is compatible with low-parametric flow cytometric analysis by applying fluorescently labeled detectors instead of metal detectors (Supplementary Fig. 4f).

The sensitivity of ACE can be further improved by applying branching amplification. After the primary cyclic extension, branching primers (a*-T-a*-**b**) were applied and crosslinked to the concatemers. Extending the branching primers through additional thermal cycles generated increased numbers of detector binding sites (Supplementary Fig. 5a). We observed that branching amplification with 50 thermal cycles generated a further ninefold signal enhancement, compared with linear amplification (Fig. 2f and Supplementary Fig. 5a). In addition, a secondary branching approach could also be applied, resulting in an additional fivefold signal increase, and a total 500-fold increase from an initial unamplified signal (Fig. 2f and Supplementary Fig. 5a). This allowed us to quantify the GFP protein expression levels in single cells as small as *Escherichia coli* (Supplementary Fig. 5b).

Taken together, these observations indicate that the ACE amplification method should enable mass cytometry to measure a broad range of low-abundance proteomic epitopes that have been challenging to assess at the single-cell resolution^9–11^.

### Multiparametric amplification using orthogonal ACE barcodes

To use ACE for simultaneous multiparametric signal amplification, we adapted our previously designed nine-mer orthogonal oligonucleotide sequence library^20^ to identify ACE initiator strands that could be extended by a corresponding set of 19-mer extender strands (Supplementary Table 1). We conjugated 37 CNVK modified detectors with metal isotope chelation polymers and validated 30 branching primers (Supplementary Table 1). As an experimental assessment of orthogonality, 33 initiator strands were labeled individually on the anti-GFP antibody (Fig. 2g). GFP-expressing HEK293T cells were stained with each of the 33 GFP antibodies in separate tubes before they were mass-tag barcoded^3,28^ and pooled for simultaneous ACE extension and detector hybridization in one tube. After the mass cytometry measurement, the degree of crosstalk was assessed by calculating the ratio between an unmatched crosstalk signal (that is, signal resulted from unmatched initiator and detectors) compared with that of a true matched signal (Fig. 2h). An average crosstalk signal of only 1.02% was observed, indicating that most of the initiator sequences could be extended and detected only by their corresponding extenders and detectors. We detected four pairs of probes with crosstalk degrees over 10% (Fig. 2h; Initiator 2–Detector 3′, Initiator 4–Detector 5*, Initiator 7–Detector 8*, Initiator 20–Detector 21*). These high-crosstalk pairs were generated between adjacent mass channels, suggesting that mass resolution issues (that is, signal spillover from the +1 or the −1 channel, an inherent artifact in mass cytometry analysis, independent of the oligonucleotide orthogonality)^29^ contributed partially to the observed crosstalk. Multiparametric ACE antibody panels were designed by considering the calculated crosstalk matrix (Supplementary Table 2) to minimize the potential influence of channel-to-channel spillovers.

### Single-cell assessment of low-abundance transcription factors

**Fig. 3:**
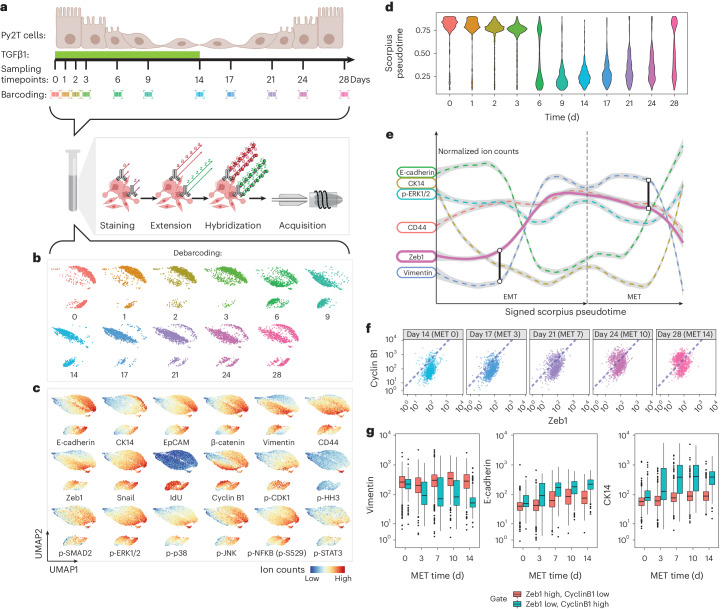
Multiplex ACE profiles molecular modulations induced by differential transcriptional factor expression levels during EMT and MET processes. **a**, Experimental workflow: mouse breast cancer Py2T cells were treated with TGFβ1 (4 ng ml^−1^) for 14 days before the stimulus was withdrawn. Cells were cultured in the absence of TGFβ1 for an additional 14 days. During the timecourse, Py2T cells were harvested on the days indicated posttreatment, barcoded and pooled into a single vial for simultaneous ACE amplification and subsequent mass cytometry analysis. Experiments were performed in biological replicates to confirm reproducibility. **b**,**c**, Dimensional reduction analysis with UMAP was performed on the data. Cells are color coded by treatment time (**b**) or abundance of measured markers after normalization (**c**) on UMAP plots. **d**, Pseudotime analysis with Scorpius was performed and plotted against the actual time posttreatment in a violin plot. **e**, Signed Scorpius analysis was used to study the molecular modulation trajectories of measured markers during the EMT–MET transitioning. **f**, Biaxial plots show the abundances of Zeb1 and cyclin B1 levels in each single cell during the MET process across the five indicated timepoints. Dashed lines indicate the gating strategy to distinguish the Zeb1 high cyclin B1 low populations and the Zeb1 low cyclin B1 high populations. **g**, Boxplots showing expression levels of a mesenchymal marker vimentin and two epithelial markers, E-cadherin and CK14, in the Zeb1 high cyclin B1 low populations and the Zeb1 low cyclin B1 high populations across the five MET timepoints. Boxplots present the first quartile (Q1), median and third quartile (Q3). The interquartile range (IQR) defines distance between Q1 and Q3. The upper whisker extends from the hinge to the largest value no further than 1.5 × IQR from the hinge. The lower whisker extends from the hinge to the smallest value no further than 1.5 × IQR of the hinge. Data beyond the end of the whiskers are plotted individually.

A 32-parameter ACE antibody panel (Supplementary Table 3) was created to profile signaling molecules, cell phenotypic markers and transcription regulators during the EMT and a reverse MET process in mouse breast cancer Py2T cells^26^. Epithelial Py2T cells were treated with 4 ng ml^−1^ transforming growth factor (TGF)β1 for 14 days to induce a mesenchymal transition, which was then followed by a 14-day timecourse after TGFβ was withdrawn, allowing cells to revert back to an epithelial state (Fig. 3a). Cells were harvested at 11 timepoints during the EMT–MET timeline, mass-tag barcoded and pooled into a single tube. Following fixation and permeabilization, labeling and amplification of the entire panel of ACE-conjugated antibodies was performed on the pooled single-cell sample and the cells were analyzed on a mass cytometer (Fig. 3a). We then performed a dimensional reduction analysis using uniform manifold approximation and projection (UMAP)^30^ to allow visualization of all cells through the EMT–MET processes in two-dimensional space (Fig. 3a–c). Cell phenotypical modulations and molecular profiles across the timeseries were assessed on the UMAP plots (Fig. 3b,c). The distributions of single cells in all 32 channels, measured over the timeseries, were shown as violin plots (Supplementary Fig. 6). ACE enabled the uncovering of molecular signatures involving low-abundance transcription factors Zeb1 and Snail/Slug during the EMT and MET transitioning process. Zeb1 expression increased sharply starting at day 6 after TGFβ1 treatment, whereas the levels of Snail/Slug showed a slow modest decline during the first 3 days with a secondary peak at 6 days during the EMT process (Fig. 3b,c and Supplementary Fig. 6). Along with transcriptional reprograming, the expression levels of epithelial markers, E-cadherin, CK14, EpCAM and β-catenin declined during the TGFβ1 treatment and increased upon TGFβ1 withdrawal. Mesenchymal markers vimentin and CD44 increased during TGFβ1 treatment, peaked at 14 and 9 days, respectively, and gradually decreased after TGFβ1 was removed (Fig. 3b,c and Supplementary Fig. 6). Interestingly, the proliferative signals from p-ERK1/2 and its downstream substrate p-p90RSK were reduced during the epithelial to mesenchymal transition, and increased again during the mesenchymal to epithelial transition, closely paralleling similar changes in EGFR expression (Fig. 3b,c and Supplementary Fig. 6).

Next, we applied an unsupervised trajectory inference method, Scorpius^31^, to reconstruct the EMT–MET in pseudotime. Comparing EMT–MET pseudotime with the actual time (Fig. 3d), we found that the molecularly defined mesenchymal phenotype started to appear at day 6, followed by the complete diminishment of the molecularly defined epithelial phenotype 9 days after TGFβ1 stimulation. During MET, cells with epithelial molecular properties expanded slowly through the 14-day timeseries while a large population retained its mesenchymal cell state, suggesting that some component of the emerging epithelial population arises in MET cells undergoing clonal expansion (Fig. 3d). The application of ACE allowed coupling the quantification of low-abundance transcription factors to other phenotypical markers. By analyzing measured markers through signed Scorpius pseudotime analysis (Methods), we confirmed that the gain of Zeb1 expression occurs during the late EMT phase in cells with downregulated CK14. Zeb1 expression was linked closely with vimentin upregulation and the quick decline of E-cadherin. During the reverse MET process, the decline of Zeb1 expression correlated with a sharp decrease in vimentin expression, which was preceded by a rise in the levels of E-cadherin (Fig. 3e). Our method enabled the discovery of an expanding population of cells over the MET timecourse with low Zeb1 and high cyclin B1 expression levels (Fig. 3f). Only these cells showed decreased vimentin, increased E-cadherin and CK14 expression levels (Fig. 3g), suggesting that suppression of Zeb1 and the gain of cyclin B1 are hallmarks for cells undergoing a MET.

Overall, these analyses demonstrate the capability of ACE to simultaneously amplify a large number of metal channels used in mass cytometry, allowing the accurate profiling of low-abundance proteomic markers for cell state transitions like EMT and MET.

### Profiling signaling network dynamics in single T lymphocytes

Systematic profiling of TCR signaling networks at single-cell resolution has been technically challenging due to the limited abundance of phosphoproteins in T lymphocytes and their small size (a T cell is 23 times smaller than a HeLa cell, on average). Most of the phosphorylation sites on TCR signaling proteins, particularly at their basal states, do not reach the detection limit of a mass cytometer or result in signals only slightly above the limit^32–34^. We investigated whether ACE could be used to address these limitations and provide a technology capable of single-cell TCR network analysis.

**Fig. 4:**
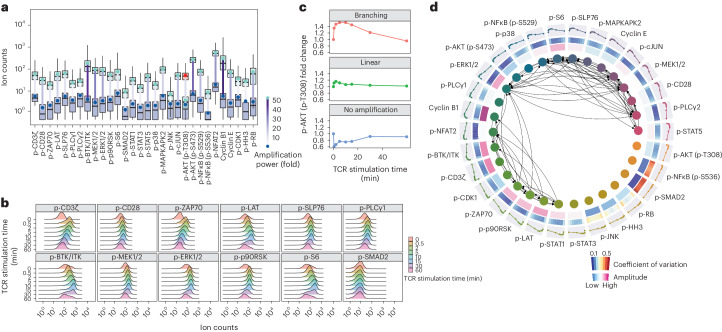
ACE enables comprehensive TCR signaling network profiling at single-cell resolution. **a**, Thirty key markers related to the TCR signaling network in the human Jurkat T cells were measured with or without ACE amplification and plotted as green or blue boxes, respectively. Mean ion counts for unamplified and linear amplified staining were indicated by the green and blue points with lines between them color coded according to amplification fold change. Boxplots present the first quartile (Q1), median and third quartile (Q3) in unamplified or linear amplified data. The IQR defines the distance between Q1 and Q3. The upper whisker extends from the hinge to the largest value no further than 1.5 × IQR from the hinge. The lower whisker extends from the hinge to the smallest value no further than 1.5 × IQR of the hinge. ACE enabled, on average, 17-fold and a maximum of 41-fold signal amplification. The signal from p-AKT (p-T308) was analyzed with an additional ACE branching amplification (shown as a red box) with 15-fold higher p-AKT (p-T308) counts after the branching amplification. **b**, Jurkat cells treated with anti-CD3 and anti-CD28 antibodies, and subsequently crosslinked with anti-mouse IgG antibody to activate TCR signaling. Cells were harvested at 0, 0.5, 2, 5, 10, 15, 30 and 60 min after TCR activation. Linear amplification allowed the quantification of key signaling nodes in the TCR network during the 1-h TCR stimulation timecourse. Negative control p-SMAD2 did not show differential phosphorylation levels as measured with ACE. **c**, Signal responses of p-AKT (p-T308) to TCR activation were analyzed without amplification (bottom), with linear amplification (middle) and with branching amplification (top). Fold changes in the p-AKT signal relative to the first timepoint (unstimulated) were calculated and showed that only with branching ACE could the signaling trajectory be captured. **d**, Circos plot shows 73 pairs of strong signaling relationships in the TCR signaling networks as detected with the BP-R^2^ method on the TCR stimulation timecourse data measured by ACE (inner layer). The middle layers show the coefficients of variation of measured phosphorylation sites at all timepoints following TCR stimulation and the signal amplitude for each phosphorylation site. The outer layer demonstrates the signaling trajectory for each marker, characterized by normalized mean ion counts across all timepoints during the TCR stimulation timecourse. Cells used in these analyses were in G1 phase to avoid cell cycle confounding effects. Experiments were performed in biological replicates to confirm reproducibility.

We created and validated a 30-parameter ACE antibody panel that included p-CD3ζ (CD247), p-CD28, p-ZAP70/SYK, p-LAT, p-SLP76, p-PLCγ1 and p-BTK/ITK in the canonical TCR pathways, p-MEK1/2, p-ERK1/2, p-p90RSK and p-S6 in the MAPK-ERK-related pathways, and many phosphorylation sites involved in stress, inflammation and cell cycle regulation (Supplementary Table 4). We first examined the ability of this multiparametric ACE antibody panel to report on TCR signaling events using human Jurkat T cells harvested over a 1-h TCR stimulation timecourse (Supplementary Fig. 7). Compared with DNA-antibody without amplification, ACE enhanced signals of measured phosphorylation sites with an average amplification power of 17 times (Fig. 4a and Supplementary Fig. 7a,b) and an increase of dynamic range by tenfold (Supplementary Fig. 7c–e). Compared with conventionally labeled mass cytometry antibodies, ACE enabled signal amplification of seven times (Supplementary Fig. 8a). For the activating phosphorylation site on AKT, phosphothreonine 308, which did not show sufficient signal (ion counts) after linear ACE amplification, we performed one round of branching ACE to further increase the detection sensitivity by 15-fold (Fig. 4a).

Over the 1-h TCR stimulation timecourse, ACE was able to reveal differential signaling responses on TCR signaling mediators (Fig. 4b and Supplementary Fig. 7b). Our data showed that phosphorylation of CD3ζ and ZAP70/SYK peaked at 0.5 min upon TCR stimulation, consistent with activation of these proteins immediately following TCR stimulation. Notably, indicators of MAPK/ERK pathway activation—p-MEK1/2, p-ERK1/2 and p-p90RSK—showed sustained signaling activities that started to decline only 30 min after TCR stimulation (Fig. 4b). As a negative control, we observed that p-SMAD2 did not respond to TCR stimulation^35^, further validating the reliability of ACE for measuring specific TCR-induced changes in signaling (Fig. 4b). By analyzing the signaling trajectory of p-AKT (p-T308) over the 1-h TCR stimulation timecourse, we found that branching ACE could uncover a ~1.5-fold signal amplitude on that phosphorylation site. The nonamplified p-AKT (p-T308) antibody lacked the sensitivity to capture the AKT signaling trajectory, which was instead dominated by technical noise (Fig. 4c). To further verify the dynamics of signaling responses captured by ACE, we compared ACE-amplified mass cytometry results with data acquired from flow cytometry using fluorescently labeled conventional antibodies. Phosphorylation levels of p-ZAP70/SYK, p-SLP76 and p-ERK1/2 in Jurkat cells were measured using both approaches and showed similar amplitudes upon stimulation (Supplementary Fig. 8b).

To systematically quantify the strength of signaling relationships in the TCR signaling networks, BP-R^2^ analysis^6^ was performed on all pairs of measured phosphorylation sites. This identified 73 strong relationships (BP-R^2^ above 0.775), which were used to reconstruct a TCR signaling network (Fig. 4d). As expected, the data recapitulated p-ERK1/2 as an essential signaling node in the TCR network, as it connects the upstream TCR responses transduced by p-ZAP70/SYK, p-SLP76 and p-MEK1/2 to downstream effectors, including p-p90RSK and p-S6 (refs. ^36,37^) (Fig. 4d). Notably, our data also showed that, following TCR engagement, most of the TCR-stimulated phosphorylation sites had a lower coefficient of variation among all of the individual cells compared with the unstimulated control or cells that had returned to steady state 1 h after TCR stimulation (Fig. 4d). This suggests a high degree of basal signaling heterogeneity, which was reduced upon TCR stimulation.

As noted from previous studies^32,33^ and reproduced in our results, signaling levels and heterogeneity in primary T lymphocytes, especially in their basal states, were more problematic to quantify with conventional mass cytometry compared to Jurkat T cells (Supplementary Fig. 8c). We used branching ACE to amplify signals of TCR-related phosphorylation sites in primary human CD4^+^ T cells harvested over a 1-h TCR stimulation timecourse and analyzed these cells with mass cytometry. Compared with the conventional approach, ACE enhanced the signals for measured sites by ten times and thus allowed characterization of signaling states and kinetics in primary human T cells (Supplementary Fig. 8c–e).

In summary, our results illustrate that ACE enabled a comprehensive analysis of TCR signaling networks at single-cell resolution, which has been previously considered challenging.

### POF samples modulate immunological signaling network responses

**Fig. 5:**
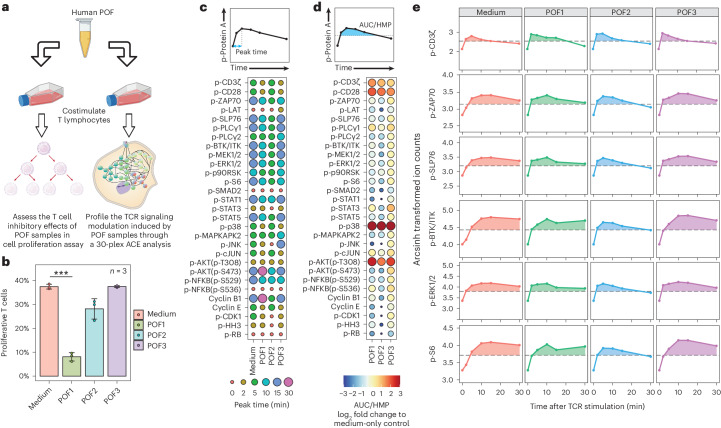
ACE characterizes T cell signaling network dynamics modulated by POF sample cocultures. **a**, Proliferation and TCR signaling effects of POF on human T lymphocytes were assessed using dye dilution and flow cytometry, and through the ACE-based 30-plex TCR signaling mass cytometry analysis, respectively. **b**, Percentage of proliferative T lymphocytes after treatment with anti-CD3/anti-CD28 beads or with indicated POF samples. Data are presented as mean values ± s.d.; *n* = 3 individual experiments. ***P < 0.001 using a two-tailed *t*-test); ***P value = 0.0000832. **c**, TCR stimulation was performed in the presence of POF or medium control. The cells were fixed 0, 2, 5, 10, 15 and 30 min later, and analyzed by ACE. The timepoint when the maximal signal for each specific phosphorylation site was observed after TCR stimulation is shown. **d**, The integrated signal corresponding to each phosphorylation site across the stimulation timecourse was calculated as the AUC/HMP. This differs across all POF treatment conditions and the medium-only control as indicated by circle size and color. **e**, Signaling trajectories for p-CD3ζ, p-ZAP70, p-SLP76, p-BTK/ITK, p-ERK1/2 and p-S6 for each POF sample and the medium-only control are shown. Dashed lines indicate HMP signals; shading defines the AUC/HMP.

Extensive tissue injury caused by trauma or major surgical procedures has long been linked to the late development of an immunosuppressive state, characterized by prolonged inflammation, refractory T cell responses and some degree of T cell death^38–40^. This phenomenon of injury-induced T cell paralysis and death appears to be induced by some combination of wound-related cytokines/chemokines and/or mononuclear cells, although the process is incompletely understood^41,42^. We applied ACE to further characterize the inhibitory regulation of T cell signaling in response to cues originating in the environment surrounding sites of tissue injury by coculturing T cells with human patient postoperative drainage fluid (POF). The immunosuppressive properties of the POF samples were first assessed using dye-based T cell proliferation assays. Signaling responses were subsequently assessed using the immune-based 30-parameter ACE protein phosphorylation panel (Fig. 5a).

We found POF1 reduced T cell proliferation, compared with the medium-only control, suggesting a highly immunosuppressive environment caused by this POF sample. POF2 inhibited T cell proliferation to a lesser degree than POF1, consistent with moderate immunosuppression, whereas POF3 did not demonstrate any T cell inhibitory effects (Fig. 5b and Supplementary Fig. 9).

We next analyzed the signaling dynamics of measured phosphorylation sites in the TCR signaling network profiling using ACE, and characterized the peak signaling times and total signaling integrals (that is, areas between the signaling trajectory (AUC) and half-maximal point (HMP); Fig. 5c–e; Methods). In most of the proximal TCR signaling and MAPK/ERK nodes, including p-ZAP70/SYK; p-SLP76; p-PLC-γ1; p-BTK/ITK; p-STAT-1, -3 and -5; p-MEK1/2; p-ERK1/2; p-p90RSK and p-S6, we observed a decrease in total signaling responses and a shift towards earlier peak times in POF1 and POF2 cotreated samples, compared with the medium-only control and POF3 cotreatment (Fig. 5c–e). Similarly, POF1 and 2 cotreatments resulted in suppression of cyclin E, cyclin B, p-CDK1 and p-HH3 compared with POF3 and the medium control. Taken together, these data indicate that POF1 and POF2 cotreatment caused a reduced and more transient TCR signaling response to anti-CD3/anti-CD28, leading to a net reduction in proliferative signals compared with the control. These findings are consistent with a requirement for sustained MAPK/ERK activation for T cell proliferation^43,44^, in good agreement with our observations from the T cell proliferation assay (Fig. 5b and Supplementary Fig. 9). Finally, these results demonstrate that ACE-based TCR signaling network profiling can be used to explore molecular mechanisms underlying immunosuppression after tissue injury at single-cell resolution, with the potential to identify targets for future therapeutic intervention.

### Signal amplification for IMC multiparametric spatial analyses

Substantial autofluorescence of renal tissue^45^ restricts the use of fluorescence-based multiparametric imaging approaches^46–48^ in the spatial profiling of proteins in histologic sections of the human kidney. IMC analysis is void of sample autofluorescence effects^7^. However, due to problems with instrument sensitivity and the lack of proper signal amplification methods, it has been reported that many antibodies showed poor signals on IMC despite their good performances in immunohistochemistry^11^. As another application of ACE, we coupled our amplification approach with IMC to demonstrate a highly sensitive multiparametric spatial analysis for human kidney protein markers.

**Fig. 6:**
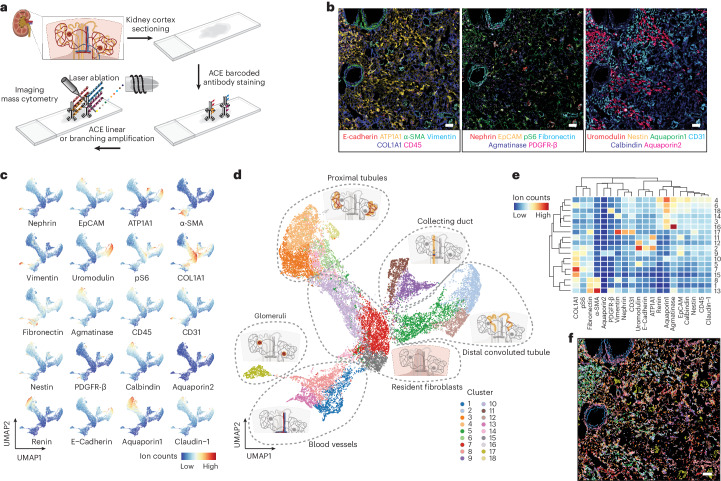
ACE enhances IMC-based multiparametric tissue profiling. **a**, Renal cortex tissue from a patient with polycystic kidney disease was used to demonstrate the application of ACE for IMC-based tissue imaging. Thermal-cycling reactions were performed on glass slides. **b**, Simultaneously measured IMC channels were overlaid to demonstrate the high specificity of ACE in spatial tissue protein profiling. **c**, After single-cell segmentation, dimensional reduction analysis with UMAP was performed to project cells on a two-dimensional space. Cells are color coded by normalized abundances of measured markers on the plots. **d**, Using the Phenograph algorithm, 18 clusters of cells were identified and color coded on the UMAP plot. These cell classifications suggested phenotypical heterogeneity in six main renal tissue compartments (encircled with dashed lines). **e**, The mean expression level of each marker was computed for each cell cluster and is shown as a heatmap. **f**, The identity of every single cell as classified by Phenograph was illustrated on the segmentation mask to generate a pseudoimage showing the kidney tissue organization by identified cell type. Experiments were performed in replicates to confirm reproducibility. Scale bar, 200 μm.

We established a 20-antibody panel, which included markers commonly used in immunohistochemistry, to assess human renal disorders (Supplementary Table 5). Validated antibodies were applied to a cryosection slide of the renal cortex from a patient with polycystic kidney disease, followed by a linear ACE amplification for all markers. We applied branching amplification to ten low-abundance kidney markers, further amplifying their signals for enhanced detection (Supplementary Fig. 10a,b and Supplementary Table 5). After the detector hybridization, signals were acquired from the samples using an IMC instrument (Fig. 6a), reconstructed as multiparametric images (Supplementary Fig. 10c), and are displayed as overlaid images (Fig. 6b). Fibronectin and α-smooth muscle actin (SMA) were observed in vesicular structures with three layers of mutually exclusive expression profiles: an inner layer of fibronectin^+^ endothelial cells and a middle layer of vesicular smooth muscle cells (α-SMA^+^) surrounded by an outer layer of fibroblasts (fibronectin^+^) (Fig. 6b and Supplementary Fig. 10c). Epithelial cells in the kidney tissue showed differential staining patterns for various markers including EpCAM, ATP1A1, uromodulin, E-cadherin, aquaporin1 and aquaporin2. The presence of the tubular segment-specific makers, aquaporin1, uromodulin or aquaporin2 identified cells in the proximal tubules, distal convoluted tubules and collecting ducts, respectively. The abundance of ATP1A1 varied across the tubular system, with stronger expression levels seen in the distal convoluted tubules and collecting ducts, compared with the levels of ATP1A1 in proximal tubules (Fig. 6b and Supplementary Fig. 10c). Vimentin, nephrin, fibronectin and CD31 were positive in clusters of cells through the section, revealing the structure of glomeruli. Nestin expression was detected with high heterogeneity in these glomeruli (Fig. 6b and Supplementary Fig. 10c). Strongly upregulated nestin levels indicate the gain of stemness in repopulating mesangial cells in kidney disorders^49–51^. The variance of glomeruli nestin levels in our sample potentially suggests different pathological stages of injury and repair within the same tissue.

To further classify and quantify the cells profiled in our ACE–IMC analysis, we performed single-cell segmentation analysis using Mesmer^52^, and embedded the segmented cells into a two-dimensional UMAP plot that was colored by the expression level of each measured maker (Fig. 6c) or by the cell clusters identified using the Phenograph algorithm^5^ (Fig. 6d). We then computed the average abundance of each marker over all identified clusters (Fig. 6e). Cluster identities were also mapped to the single-cell mask to generate a pseudoimage colored by detected cell types (Fig. 6f). These analyses identified the glomerular compartment within the analyzed kidney section consisting of cells in cluster 17, with a subpopulation showing high nestin expression levels—a potential biomarker of mesangial expansion in this patient with polycystic kidney disease (Fig. 6c,d). The blood vessel compartment was identified by the presence of endothelial cells and smooth muscle cells, indicated by clusters 1 and 13, respectively (Fig. 6c,d). In addition, our analyses delineated proximal tubules, distal convoluted tubules and collecting ducts in the renal tubular system that were well distinguished on the UMAP plot (Fig. 6d). Marker expression profiles varied within these tissue compartment (Fig. 6c,e), indicating tissue heterogeneity that could potentially lead to more defined cell type classification. Finally, a resident fibroblast compartment with high COL1A1 expression levels was also detected in our analysis (Fig. 6c,d and Supplementary Fig. 10c).

Overall, our results demonstrate the application of ACE to IMC-based multiparametric tissue imaging with enhanced sensitivity. We recapitulated known tissue compartments in the human kidney and revealed various spatial aspects of the pathological state in diseased human tissue.

## Discussion

Here, we present ACE, which enables signal amplification in mass cytometry-based single-cell and spatial tissue analysis. ACE tags proteins of interest in single cells or tissue samples using antibodies conjugated with DNA barcodes, which in turn undergo repeated in situ extension under thermal-cycling conditions to generate long concatemers that allow hybridization and crosslinking of hundreds of detectors on each antibody through CNVK-based ultrasensitive nucleic acid photocrosslinking. ACE showed superior efficiency (over 500 times signal amplification through branching), controllability, multiplexity and signal-to-noise ratio, compared with conventional signal amplification approaches^14,18,21^. We demonstrated the high orthogonality of ACE probe sequences, which was verified in two independent multiparametric antibody panels, both with over 30 targets. Our results validated the compatibility of ACE for use in conjugation with mass-tag cellular barcoding, iridium DNA intercalation and regular mass cytometry antibody staining protocols. Finally, we applied ACE in high-sensitivity tissue profiling using IMC and showed that our approach is capable of characterizing pathological states in the human kidney under conditions where autofluorescence limits the use of conventional fluorescence-based imaging approaches^45^.

The ACE platform expands the coverage of mass cytometry analysis by allowing challenging questions in the realm of the low-abundance proteome to be addressed. Previously undetectable low-abundance markers, such as many transcription factors and rare phosphorylation sites, can now be quantified with enhanced sensitivity at single-cell resolution. In addition, ACE allows single-cell characterization of immune signaling responses, which has previously been considered problematic to study at single-cell resolution^9,10,32,34^. Here, we showed a potential implementation of ACE in estimating the degree of immunosuppression in patient POF samples through high-profile single-cell TCR network dynamic analysis. The use of ACE may also allow an improvement in the spatial resolution of IMC, particularly under conditions where signal loss resulting from reducing the laser ablation crater size can be compensated by the amplified ion abundances from a specific ACE-examined marker.

We noted a few limitations in ACE to be improved with future work. The current ACE reaction requires using detergent Triton X-100 to permeabilize cells. Cell surface proteins have to be stained with compatible protocols before ACE-based signal amplification (Supplementary Fig. 2a). Analysis of the low-abundance proteome is limited by current antibody availability. Low signal-to-noise ratio caused by poor antibody specificity cannot be improved by applying ACE. Nevertheless, for intracellular markers, particularly transcription factors and signaling proteins for which good antibodies exist, ACE has demonstrated substantially increased sensitivity and accuracy, compared with conventional mass cytometry approaches. Our approach is particularly useful for low-abundance markers that are barely detectable or undetectable in conventional mass cytometric assays.

## Methods

### Cell culture

HEK293T cells, obtained from ATCC (CRL-3216), and Py2T cells, a gift from G. Christofori at the University of Basel^26^, were cultured in DMEM (Sigma, cat. no. D5671), supplemented with 10% FBS, 2 mM l-glutamine, 100 U ml^−1^ penicillin and 100 µg ml^−1^ streptomycin. For cell passaging or harvesting, cells were incubated with 1× TrypLE Express (Life Technologies) for 2 min (HEK293T) or 10 min (Py2T) at 37 °C. Jurkat cells, obtained from ATCC (TIB-152), were cultured in RPMI-1640 medium (Gibco, cat. no. 52400025) supplemented with 10% FBS, 100 U ml^−1^ penicillin and 100 µg ml^−1^ streptomycin.

### Transfection

HEK293T cells were seeded at a density of 0.7 million per well in six-well plates. After 24 h, cells were transfected with 2 μg plasmid encoding ERK2–GFP^53^ and 4 μl of jetPRIME (PolyPlus) per well with the standard protocol provided by the manufacturer.

### Inducing EMT and MET with TGFβ1 stimulation

Py2T cells were seeded at 15% confluency and cultured in medium containing 4 ng ml^−1^ recombinant human TGFβ1 (BioLegend) for 14 days before TGFβ1 was withdrawn and cells were cultured continuously in regular medium for an additional 14 days. During the timecourse, Py2T cells were harvested on days 0, 1, 2, 3, 6, 9, 14, 17, 21, 24 and 28. For cell passaging and harvesting, the medium was replaced with 1× TrypLE to induce cell detachment. Paraformaldehyde (PFA, Electron Microscopy Sciences) was added to a final percentage of 1.6% for a 10-min incubation at room temperature (RT) to fix cells in their suspension. Crosslinked cells were washed twice with cell staining medium (CSM; PBS with 0.5% BSA, 0.02% NaN_3_) and, after centrifugation, ice-cold methanol was used to resuspend the cells, followed by a 10-min permeabilization on ice for long-term storage at −80 °C.

### TCR stimulation

Jurkat cells or primary human CD4^+^ T lymphocytes were spun down and resuspended in 200 μl 4 °C RPMI medium and incubated with anti-CD3 (Clone UCHT1 from BD Biosciences; 0.2 μg per 10^6^ cells) and anti-CD28 (Clone CD28.2 from BD Biosciences; 0.2 μg per 10^6^ cells) antibodies for 15 min on ice. Cells were then washed once and resuspended in 100 μl RPMI medium for 5 min incubation at 37 °C before the anti-mouse Ig antibody (Polyclonal Goat Anti-Mouse Ig from BD Biosciences, 0.4 μg per 10^6^ cells) was added to crosslink the anti-CD3 and anti-CD28 antibodies. For POF sample treatment, the anti-mouse Ig antibody was diluted directly in POF samples before the mixture was added to cells. Cells were then kept at 37 °C for 0, 0.5, 2, 5, 15, 30 or 60 min before 1.6% PFA was used for a 10-min fixation at RT. Cells were then washed and permeabilized with 100% ice-cold methanol for 10 min for long-term storage at −80 °C.

### T cell proliferation assays

In the T cell proliferation assay, primary T lymphocytes were isolated from peripheral blood of healthy donors using human Pan T Cell negative Isolation Kit (Miltenyi Biotec). Isolated T cells were labeled at the concentration of 5 × 10^6 ^cells ml^−1^ with 5 µM CellTrace Violet (Thermo Fisher). T cells were then stimulated with Dynabeads Human T-Activator CD3/CD28 (Thermo Fisher) at a ratio of 3.75 µl beads per 150,000 cells and cultured in the presence of one of three individual POF samples at a 1:2 dilution with RPMI medium, or with a RPMI medium-only control. Cell proliferation was assessed 4 days later by flow cytometry measurements of CellTrace Violet dye dilution. All conditions of experiments were performed with three individual sample replicates.

### Antibody conjugation

We have described the oligonucleotide–antibody conjugation method elsewhere^21^. In brief, initiators were ordered through IDT as 5′-thiolated oligonucleotides and were dissolved in IDTE (pH 8.0) at 1 mM concentration (Supplementary Table 1). For each conjugation, 10 nmol oligonucleotides (10 μl from the stock) were reduced in PBS containing 100 mM TCEP (Sigma-Aldrich) and 0.55 mM EDTA at 37 °C for 1 h, followed by washing five times with PBS in a 3-kDa Amicon filter to remove the TCEP. Antibodies were washed twice in a 50-kDa Amicon filter before five times excessive amount of SM(PEG)_2_ crosslinker (Thermo Fisher) was added directly to the filter for a 2-h incubation at 4 °C. Antibodies were then washed three times with PBS. Next, reduced oligonucleotides were added to the 50-kDa Amicon filter containing SM(PEG)_2_-modified antibodies at a 5:1 (oligonucleotide:antibody) molar ratio. The mixture was kept at 4 °C overnight and was washed five times the next day with PBS to remove excessive oligonucleotides. Antibody–oligonucleotide conjugates in the residue volume were collected and the antibody concentration was measured using Qubit protein assay (Thermo Fisher). At least 50% of Candor PBS Antibody Stabilization solution (Candor Bioscience GmbH) was used to dilute antibodies for long-term storage at 4 °C. Antibodies used in this study were listed in Supplementary Tables 3–5. Antibodies were validated thoroughly by the venders and in previous studies^6,19,21,54,55^.

### Detection oligonucleotide conjugation

The detector was ordered from GeneLink with an internal CNVK modification, a 5′-end thiol modification and an optional 3′-end Cy5 modification for flow cytometry verification experiments (Supplementary Table 1). The detector conjugation protocol was adapted from ref. ^56^; 5 nmol detection oligonucleotides were incubated with 50 mM TCEP in a total volume of 50 μl at RT for 30 min. Ethanol precipitation was then performed on reduced detectors, in which 140 μl water, 20 μl 3 M sodium acetate (pH 5.3) and 600 μl pure ethanol were added to the oligonucleotide mixture before it was transferred to a −80 °C freezer for overnight precipitation. On the second day, the oligonucleotide was taken from the −80 °C freezer and centrifuged at 14,000*g* for 30 min at 4 °C. We then washed the oligonucleotide pellet in ice-cold 75% ethanol before it was centrifuged at 14,000*g* for another 10 min at 4 °C. Next, the pellet was air dried and subsequently dissolved in 100 μl C buffer (Standard BioTools Maxpar X8 Antibody Labeling Kit). Nanodrop was used to determine the oligonucleotide concentration. In parallel, a tube of DTPA polymer was taken from the Standard BioTools Maxpar X8 Antibody Labeling Kit; 95 μl L buffer and 5 μl isotope solution were added to the polymer before an incubation at 37 °C for 30 min. The mixture was then transferred to a 3-kDa Amicon filter, supplemented with 200 μl L buffer, and centrifuged at 12,000*g* for 25 min at RT; 300 μl C buffer was used to wash the metal polymer in a 30-min 12,000*g* centrifugation step at RT. Next, the reduced oligonucleotide and the chelated metal polymer were mixed in an Eppendorf tube for a 2-h incubation at RT before TCEP was added to the final concentration of 5 mM for an additional incubation for 30 min. Finally, the conjugated detectors were washed twice with ddH_2_O in a 30-kDa Amicon filter before determining the oligonucleotide concentration on a nanodrop. ddH_2_O was used to dilute the oligonucleotide to 10 μM for 4 °C (short-term) or −20 °C (long-term) storage.

### Mass-tag barcoding, antibody staining and ACE amplification for single-cell suspensions

PFA-crosslinked and methanol-permeabilized cells were washed three times with CSM and once with PBS. Cells were incubated in PBS containing barcoding reagents (^102^Pd, ^104^Pd, ^105^Pd, ^106^Pd, ^108^Pd, ^110^Pd, ^113^In and ^115^In) at a final concentration of 100 nM for 30 min at RT and then washed three times with CSM^3^. Barcoded cells were then pooled and stained with the oligonucleotide-conjugated antibody mix (Supplementary Tables 3 and 4) in CSM containing 0.5% dextran sulfate and 4 mM EDTA at RT for 1 h. The antibody mixture was removed by washing cells three times with CSM and once with PBST (0.1% Triton X-100 dissolved in PBS) before 5 mM BS(PEG)_5_ crosslinker (Thermo Fisher) in PBST was applied to crosslink the antibodies for 10 min. Subsequently, the crosslinker was quenched in a 5-min incubation with 0.1 M glycine in PBST and cells were further washed twice with PBST. Next, an extension mixture was prepared that contained 10% (v/v) ThermoPol Reaction Buffer (NEB), 0.2 mM dA/C/TTP (NEB), 1 μM extender strands (Supplementary Table 1), 1,200 units ml^−1^ *Bst* DNA Polymerase (NEB). Cell pellets were resuspended in the extension mix and redistributed into PCR tubes with a maximal volume of 75 μl for each tube. The extension reaction was then performed on a thermal cycler with desired number of cycles (500 for the experiment in Fig. 2b, 100 for all other ACE experiments in cell suspensions) of extension (22 °C for 40 s) and denaturing (58 °C for 20 s). Next, cells were taken from the thermal cycler and washed with PBST three times at RT and an additional three times at 58 °C. Detectors or branching primers were diluted in PBST at final concentrations of 100 nM before being added to the cells for a 30-min incubation at RT. Cells were then washed with PBST three times at RT and once at 37 °C before being exposed to 365-nm UV for 5 s under a LED UV flashlight (Electro-Lite LED-200). Cells were further washed with PBST three times at RT and once at 58 °C to remove uncrosslinked detection or branching primers. For experiments involving branching, cells were then resuspended in extension mixture and amplified through 50 thermal cycles before the detectors were applied. In the experiments performed in Fig. 2b and Fig. 2f, conventional secondary antibodies conjugated directly with the Maxpar X8 Antibody Labeling Kit (Standard BioTools) were applied to the cells that had already undergone ACE thermal cycling. After a 1-h incubation at RT, cells were washed three times in CSM and once in PBST to remove the excessive secondary antibodies. Finally, DNA staining was performed: iridium-containing intercalator (100 nM, Standard BioTools) diluted in PBS with 1.6% PFA was incubated with the cells at 4 °C overnight. On the day of measurement, the intercalator solution was removed and cells were washed with CSM, PBS and ddH_2_O. After the last washing step, cells were resuspended in ddH_2_O and filtered through a 70-μm strainer.

### *E. coli* staining and ACE amplification

The wild-type *E. coli* DH5α strain or the GFP-expressing *E. coli* (25922GFP, ATCC) strain were centrifuged at 7,000*g* and resuspended in 4% PFA for a 10-min fixation. *E. coli* cells were washed three times with PBS before being permeabilized in 70% ethanol for 1 h at RT and washed three times with PBS. Cell walls were partially digested by an incubation in 25 μg ml^−1^ lysozyme in TEG buffer (25 mM Tris-HCl, 10 mM EDTA and 50 mM glucose) for 10 min at RT and washed three times with PBS. CSM was used as a blocking agent for 30 min before the oligonucleotide-conjugated anti-GFP antibody was applied to the cells in PBS containing 0.5% dextran sulfate and 4 μM EDTA for a 1-h incubation. Cells were washed three times in CSM and once with PBST before being fixed with 5 mM BS(PEG)_5_ for 30 min then quenched with 0.1 M glycine. Branching amplification was performed using the No. 29–1 and No. 1–20 branching oligos (Supplementary Table 1) using protocols described above before the detector 20* strand was added and crosslinked after UV exposure. As in the mammalian cell staining, iridium-containing intercalator (100 nM, Standard BioTools) in PBS with 1.6% PFA was incubated with the cells at 4 °C overnight. On the day of measurement, cells were washed with CSM, PBS and ddH_2_O before being resuspended in ddH_2_O and filtered through a 70-μm strainer.

### Suspension mass cytometry analysis

EQ Four Element Calibration Beads (Standard BioTools) were added to cell suspensions at a 1:10 ratio (v/v). Samples were analyzed on a Helios (Standard BioTools) connected to a Super Sampler (Victorian Airship). The manufacturer’s standard operation procedures were used for acquisition at a cell rate of ~100 cells per second. CyTOF Software (Standard BioTools) was used for mass cytometry data collection. After acquisition, all FCS files from the same barcoded sample were concatenated^3^. Data were then normalized and bead events were removed^57^ before doublet removal and debarcoding of cells into their corresponding wells using a doublet-filtering scheme and single-cell deconvolution algorithm^28^. Subsequently, data were processed using Cytobank (http://www.cytobank.org/). Additional gating on the DNA channels (^191^Ir and ^193^Ir) was used to remove remained doublets, debris and contaminating particulates.

### ACE amplification on tissue slide and IMC analysis

Human kidney cryosection tissue slides were purchased from OriGene (Sample ID: FR0002ED32). On the day of experiment, the cryosection slides were rinsed briefly with PBS before the tissues were crosslinked with 4% PFA during a 15-min incubation. Slides were then rinsed again with PBS and incubated with the blocking/permeabilization buffer (PBS with 5% BSA and 0.3% Triton X-100) for 30 min. The oligonucleotide-conjugated antibody mixture (Supplementary Table 5) was prepared by diluting antibodies in the blocking/permeabilization buffer supplemented with 0.02% dextran sulfate and 4 mM EDTA. The mixture was applied on tissue samples for an overnight incubation at 4 °C. On the next day, the antibody mixture was removed by three 5-min washing steps with PBST before 5 mM BS(PEG)_5_ crosslinker (diluted in PBST) was applied for a 10-min incubation to crosslink the antibodies. Subsequently, the crosslinker was quenched by 0.1 M Glycine in PBST for 5 min. Tissues were further washed three times (5 min each) with 60% formamide in PBST and rinsed once with PBST. An extension mixture was prepared that contained 10% (v/v) ThermoPol Reaction Buffer (NEB), 0.2 mM dA/C/TTP (NEB), 1 μM extender strands (Supplementary Table 1), 1,200 units ml^−1^ *Bst* DNA Polymerase (NEB). A removable 3-well silicone chamber (80381, ibidi) was transferred on each tissue slide and pressed with force to ensure tight adhesion to the slide. Extension mixture (200 μl) was added to each tissue sample encircled by the silicone gasket before the lid of the original silicone chamber was applied. The extension reaction was then performed on a flat-top thermal cycler (Eppendorf Mastercycler Nexus Flat) with the desired number of cycles (220 for the experiments in Fig. 6) of extension (22 °C for 70 s) and denaturing (58 °C for 60 s). Next, slides were taken from the thermal cycler, washed three times (5 min each) with 60% formamide in PBST and rinsed once with PBST. Branching primers were diluted to final concentrations of 500 nM in 2× SSCT containing 10% dextran sulfate and 30% formamide before being added to the tissue slides for a 30-min incubation at RT. Samples were then washed with PBST containing 30% formamide three times at RT before being exposed at 365-nm UV for 5 s under a LED UV flashlight (Electro-Lite LED-200). An extension mixture was prepared and added to the samples to extend the branching oligos with the desired number of cycles (220 for the experiments in Fig. 6) using the same conditions as in the primary extension. After thermal cycling, slides were washed three times (5 min each) with 60% formamide in PBST and rinsed once with PBST. Detectors were diluted in PBST to final concentrations of 200 nM before being added to the slides for a 30-min incubation at RT. Tissues were then washed with PBST three times at RT before being exposed at 365-nm UV for 5 s. Finally, DNA staining was performed; iridium-containing intercalator (500 nM, Standard BioTools) diluted in PBST was applied to the tissues for a 30-min incubation followed by washing three times with PBST and a brief rinse with ddH_2_O. The samples were left to dry and stored at RT before images were acquired using a Hyperion XTi Imaging System (Standard BioTools) at the ablation frequency of 800 Hz. CyTOF Software (Standard BioTools) was used for imaging mass cytometry data collection.

### Quantification and statistical analysis

#### Suspension mass cytometry data preprocessing

Raw ion counts were transformed using the inverse hyperbolic sine transform with a cofactor of 5:

$${\rm{data}}={\rm{arcsinh}}({\rm{dataraw}}/5)$$

Except where use of raw data values is specifically noted, all visualizations and analyses were performed using transformed data.

#### IMC data preprocessing and single-cell segmentation

Single-cell segmentation was performed on IMC images using the DeepCell Mesmer machine learning-based segmentation model^52^. DNA intercalator ions ^191^Ir and ^193^Ir were used to indicate the nuclei, whereas aquaporin1, aquaporin2, ATP1A1, α-SMA, vimentin and uromodulin were used as cytoplasmic segmentation markers. Segmented single-cell masks were imported into CellProfiler^58^ where objects with fewer than 30 pixels were removed and ion counts of all pixels in a segmented cell were averaged for each analyzed marker.

#### UMAP analysis

UMAP analysis^30,59^ was performed with the Package ‘umap’ in R. Parameters used for the analysis in Fig. 3b,c are listed as follows: n_neighbors = 100, min_dist = 0.4. Parameters used for the analysis in Fig. 6c,d are listed as follows: n_neighbors = 10, min_dist = 0.1.

#### Scorpius analysis

Scorpius^31^ pseudotime reconstruction was performed with the Package ‘SCORPIUS’ in R. Signed Scorpius was done by first reconstructing the pseudotime using all cells regardless of EMT or MET status and then assigning cells in the first 14 days of treatment (EMT cells) as negative in the pseudotime.

#### BP-R^2^

BP-R^2^ analysis^6^ was used as a measure of the strength of signaling relationships. The threshold of BP-R^2^ score to determine a strong relationship is a sum of 6.2 over the eight TCR stimulation timepoints, or an average BP-R^2^ score of 0.775 for each pair of measured phosphorylation sites.

#### Signaling integral analysis

In the signaling integral measurement in Fig. 5d, the mean ion counts for each measured phosphorylation site over the timecourse were first calculated, as presented in the signaling trajectories in Fig. 5e. The highest mean ion counts across all treatment condition over the timecourse was divided by two to calculate a HMP. Signal integral was then computed as the size of the area under the signaling trajectory and above the HMP level using trapezoid integration.

#### Phenograph clustering

Cell clustering was performed with the Phenograph algorithm^5^ using the Package ‘Rphenograph’ in R. All analyzed markers were used to generate the Phenograph clusters and the parameter of nearest neighbors was set as 20.

### Reporting summary

Further information on research design is available in the Nature Portfolio Reporting Summary linked to this article.

## Online content

Any methods, additional references, Nature Portfolio reporting summaries, source data, extended data, supplementary information, acknowledgements, peer review information; details of author contributions and competing interests; and statements of data and code availability are available at 10.1038/s41587-024-02316-x.

## Supplementary information

Supplementary Information Supplementary Figs. 1–10.

Reporting Summary

Supplementary Tables 1–5. Table 1. ACE sequence list; Table 2. ACE sequence crosstalk ratios; Table 3. Antibody panel EMT; Table 4. Antibody panel TCR signaling; Table 5. Antibody panel kidney cortex tissue.

## Data Availability

All raw data are available at https://community.cytobank.org/cytobank/projects/1561 (ref. ^60^).

## Code availability

Code is available on GitHub at https://github.com/xiaokanglun/ACE (ref. ^61^).
