# Supplementary material for: Signal amplification by cyclic extension enables high-sensitivity single-cell mass cytometry
Source: Nat Biotechnol. 2024 Jul 29;43(5):811–21. doi: 10.1038/s41587-024-02316-x (PMC11910986; doi:10.1038/s41587-024-02316-x)
Supplement: Supplementary file 2 — Reporting Summary [file 41587_2024_2316_MOESM2_ESM.pdf]

nature portfolio

Corresponding author(s): Peng Yin

Last updated by author(s): Apr 8, 2024

Reporting Summary

Nature Portfolio wishes to improve the reproducibility of the work that we publish. This form provides structure for consistency and transparency in reporting. For further information on Nature Portfolio policies, see our Editorial Policies and the Editorial Policy Checklist.

Statistics

For all statistical analyses, confirm that the following items are present in the figure legend, table legend, main text, or Methods section.

n/a	Confirmed	
☐	☒	The exact sample size (*n*) for each experimental group/condition, given as a discrete number and unit of measurement
☐	☒	A statement on whether measurements were taken from distinct samples or whether the same sample was measured repeatedly
☐	☒	The statistical test(s) used AND whether they are one- or two-sided<br>*Only common tests should be described solely by name; describe more complex techniques in the Methods section.*
☐	☒	A description of all covariates tested
☒	☐	A description of any assumptions or corrections, such as tests of normality and adjustment for multiple comparisons
☐	☒	A full description of the statistical parameters including central tendency (e.g. means) or other basic estimates (e.g. regression coefficient) AND variation (e.g. standard deviation) or associated estimates of uncertainty (e.g. confidence intervals)
☐	☒	For null hypothesis testing, the test statistic (e.g. *F*, *t*, *r*) with confidence intervals, effect sizes, degrees of freedom and *P* value noted<br>*Give P values as exact values whenever suitable.*
☒	☐	For Bayesian analysis, information on the choice of priors and Markov chain Monte Carlo settings
☒	☐	For hierarchical and complex designs, identification of the appropriate level for tests and full reporting of outcomes
☐	☒	Estimates of effect sizes (e.g. Cohen's *d*, Pearson's *r*), indicating how they were calculated

Our web collection on statistics for biologists contains articles on many of the points above.

Software and code

Policy information about availability of computer code

Data collection	The CyTOF Software (v7.0.8493) from Standard BioTools was used for suspension mass cytometry data collection and de-convolution. The CyTOF Software (v9.0) from Standard BioTools was used for imaging mass cytometry data collection. The FACSDiva Software was used for flow cytometry data collection.
Data analysis	Data preprocessing was completed using www.cytobank.org. Data analysis and visualization were performed using R 4.0.5. Additional package for the UMAP, Scorpius, Phenograph, and BP-R2 analyses were described in the Methods section. Code is available on GitHub at https://github.com/xiaokanglun/ACE.

For manuscripts utilizing custom algorithms or software that are central to the research but not yet described in published literature, software must be made available to editors and reviewers. We strongly encourage code deposition in a community repository (e.g. GitHub). See the Nature Portfolio guidelines for submitting code & software for further information.

Data

Policy information about availability of data

All manuscripts must include a data availability statement. This statement should provide the following information, where applicable:

- Accession codes, unique identifiers, or web links for publicly available datasets
- A description of any restrictions on data availability
- For clinical datasets or third party data, please ensure that the statement adheres to our policy

All raw data are available at community.cytobank.org/cytobank/projects/1561.

Human research participants

Policy information about studies involving human research participants and Sex and Gender in Research.

Reporting on sex and gender	N.A.
Population characteristics	N.A.
Recruitment	N.A.
Ethics oversight	N.A.

Note that full information on the approval of the study protocol must also be provided in the manuscript.

Field-specific reporting

Please select the one below that is the best fit for your research. If you are not sure, read the appropriate sections before making your selection.

☒ Life sciences ☐ Behavioural & social sciences ☐ Ecological, evolutionary & environmental sciences

For a reference copy of the document with all sections, see nature.com/documents/nr-reporting-summary-flat.pdf

Life sciences study design

All studies must disclose on these points even when the disclosure is negative.

Sample size	Sample size in the POF study was chosen based on statistical requirements (2-tailed t-test) to ensure robustness and reliability of our findings.
Data exclusions	No data were excluded from the analyses.
Replication	All findings have been reproduced in this study. To calculate statistical significance, three individual sample replicates were used.
Randomization	Randomization was not performed. In mass cytometry experiments, all samples were barcoded and pooled into a single tube to be measured simultaneously.
Blinding	Blinding was not performed. All data reported are quantitative.

Reporting for specific materials, systems and methods

We require information from authors about some types of materials, experimental systems and methods used in many studies. Here, indicate whether each material, system or method listed is relevant to your study. If you are not sure if a list item applies to your research, read the appropriate section before selecting a response.

Materials & experimental systems

n/a	Involved in the study
☐	☒ Antibodies
☐	☒ Eukaryotic cell lines
☒	☐ Palaeontology and archaeology
☒	☐ Animals and other organisms
☒	☐ Clinical data
☒	☐ Dual use research of concern

Methods

n/a	Involved in the study
☒	☐ ChIP-seq
☐	☒ Flow cytometry
☒	☐ MRI-based neuroimaging

Antibodies

Antibodies used

Details of antibodies, including vendors, clone IDs, catalog numbers, and concentrations are given in Supplementary Table 3-5.

Validation

All antibodies have been throughly validated by the vendors and in our previous studies Lun et al., 2017 (doi.org/10.1038/nbt.3770), Rapsomaniki et al., 2018 (doi.org/10.1038/s41467-018-03005-5), and Lun et al., 2019 (doi.org/10.1038/s41467-018-03005-5).

Please see vendors' validation information through links below:
agmatinase Polyclonal NBP1-82080 Novus https://www.novusbio.com/products/agmat-antibody_nbp1-82080
aquaporin1 1/22 sc-32737 Santa Cruz https://www.scbt.com/p/aqp1-antibody-1-22
aquaporin2 EPR21080 ab230170 Abcam https://www.abcam.com/products/primary-antibodies/aquaporin-2-antibody-epr21080-bsa-and-azide-free-ab230170.html
ATP1A1 A19182B 604752 Biolegend https://www.biolegend.com/en-us/products/purified-anti-atp1a1-antibody-21984
calbindin D1I4Q 46343SF CST https://www.cellsignal.com/products/primary-antibodies/calbindin-d1i4q-xp-rabbit-mab-bsa-and-azide-free/46343
CD31 WM59 303102 Biolegend https://www.biolegend.com/en-us/products/purified-anti-human-cd31-antibody-883
CD44 IM7 103002 Biolegend https://www.biolegend.com/en-us/products/purified-anti-mouse-human-cd44-antibody-318
CD45 HI30 304002 Biolegend https://www.biolegend.com/en-us/products/purified-anti-human-cd45-antibody-710
CK14 Poly9060 906004 Biolegend https://www.biolegend.com/en-us/products/purified-anti-keratin-14-antibody-13379
claudin-1 EPRR18871 ab238949 Abcam https://www.abcam.com/products/primary-antibodies/claudin-1-antibody-eprr18871-bsa-and-azide-free-ab238949.html
COL1A1 E8F4L 81375SF CST https://www.cellsignal.com/products/primary-antibodies/col1a1-e8f4l-xp-rabbit-mab-bsa-and-azide-free/81375
cyclin B1 GNS-11 554178 BD https://www.bdbiosciences.com/en-us/products/reagents/flow-cytometry-reagents/research-reagents/single-color-antibodies-ruo/purified-mouse-anti-cyclin-b1.554178
cyclinE HE12 4129BF CST https://www.cellsignal.com/products/4129/applications?index=1&application=all
E-Cadherin 36/Ecadherin 610182 BD https://www.bdbiosciences.com/en-us/products/reagents/microscopy-imaging-reagents/immunofluorescence-reagents/purified-mouse-anti-e-cadherin.610182
EGFR EP38Y ab272293 Abcam https://www.abcam.com/products/primary-antibodies/egfr-antibody-ep38y-bsa-and-azide-free-ab272293.html
EpCAM G8.8 118201 Biolegend https://www.biolegend.com/en-us/products/purified-anti-mouse-cd326-ep-cam-antibody-4724
fibronectin 10/fibronectin 610078 BD https://www.bdbiosciences.com/en-us/products/reagents/flow-cytometry-reagents/research-reagents/single-color-antibodies-ruo/purified-mouse-anti-fibronectin.610078
nephrin Polyclonal AF4269 R&D https://www.rndsystems.com/products/human-nephrin-antibody_af4269
nestin 10C2 656802 Biolegend https://www.biolegend.com/en-us/products/purified-anti-nestin-antibody-8836
p-AKT pS473 D9E 4060BF CST https://www.cellsignal.com/products/primary-antibodies/phospho-akt-ser473-d9e-xp-rabbit-mab/4060
p-AKT pT308 D25E6 80722SF CST https://www.cellsignal.com/products/primary-antibodies/phospho-akt-thr308-d25e6-xp-rabbit-mab-bsa-and-azide-free/80722
p-BTK/p-ITK pY551 24a/BTK 558034 BD https://www.bdbiosciences.com/en-us/products/reagents/western-blotting-and-molecular-reagents/western-blot-reagents/purified-mouse-anti-btk-py551-itk-py511.558034
p-CD247 pY142 K25-407.69 K25-407.69 BD https://www.bdbiosciences.com/en-us/products/reagents/western-blotting-and-molecular-reagents/western-blot-reagents/purified-mouse-anti-cd247-py142.558402
p-CDK1 pY15 44 612306 BD https://www.bdbiosciences.com/en-us/products/reagents/microscopy-imaging-reagents/immunofluorescence-reagents/purified-mouse-anti-cdk1-cdc2-py15.612306
p-cJUN pS73 D47G9 77955SF CST https://www.cellsignal.com/products/primary-antibodies/phospho-c-jun-ser73-d47g9-xp-rabbit-mab-bsa-and-azide-free/77955
p-ERK pT202/pY204 20A 612359 BD https://www.bdbiosciences.com/en-us/products/reagents/flow-cytometry-reagents/research-reagents/single-color-antibodies-ruo/purified-mouse-anti-erk1-2-pt202-py204.612359
p-HH3 pS28 HTA28 641002 Biolegend https://www.biolegend.com/en-us/products/purified-anti-histone-h3-phosphorylated-ser28-antibody-5169
p-JNK pT183/Y185 G9 9255BF CST https://www.cellsignal.com/products/primary-antibodies/phospho-sapk-jnk-thr183-tyr185-g9-mouse-mab/9255
p-LAT pY226 J96-1238.58.93 J96-1238.58.93 BD https://www.bdbiosciences.com/en-us/products/reagents/microscopy-imaging-reagents/immunohistochemistry-reagents/purified-mouse-anti-lat-py226.558363
p-MAPKAPK2 pT334 27B7 3007BF CST https://www.cellsignal.com/products/primary-antibodies/phospho-mapkapk-2-thr334-27b7-rabbit-mab/3007
p-MEK pS221 166F8 75262SF CST https://www.cellsignal.com/products/primary-antibodies/phospho-mek1-2-ser221-166f8-rabbit-mab-bsa-and-azide-free/75262
p-NFAT2 pS237 EPR2377(N) ab240262 Abcam https://www.abcam.com/products/primary-antibodies/nfat2-phospho-s237-antibody-epr2377n-bsa-and-azide-free-ab240262.html

p-NFfκB pS536 93H1 3033BF CST https://www.cellsignal.com/products/primary-antibodies/phospho-nf-kb-p65-ser536-93h1-rabbit-mab/3033
p-NFκB pS529 K10-895.12.50 558393 BD https://www.bdbiosciences.com/en-us/products/reagents/flow-cytometry-reagents/research-reagents/single-color-antibodies-ruo/purified-mouse-anti-nf-b-p65-ps529.558393
p-p38 pT180/pY182 36/p38 612289 BD https://www.bdbiosciences.com/en-us/products/reagents/flow-cytometry-reagents/research-reagents/single-color-antibodies-ruo/purified-mouse-anti-p38-mapk-pt180-py182.612289
p-p90RSK pS380 D5D8 12032BF CST https://www.cellsignal.com/products/antibody-conjugates/c-jun-60a8-rabbit-mab-alexa-fluor-555-conjugate/31032
p-PLCγ1 pY783 A17025A 612402 Biolegend https://www.biolegend.com/en-us/products/purified-anti-plcgamma1-phospho-tyr783-antibody-17738
p-PLCγ2 pY759 E9E9Y 50535BF CST https://www.cellsignal.com/products/primary-antibodies/phospho-plcg2-tyr759-e9e9y-rabbit-mab/50535
p-RB S807/811 D20B12 8516BF CST https://www.cellsignal.com/products/primary-antibodies/phospho-rb-ser807-811-d20b12-xp-rabbit-mab/8516
p-S6 pS235/236 D57.2.2E 4858BF CST https://www.cellsignal.com/products/primary-antibodies/phospho-s6-ribosomal-protein-ser235-236-d57-2-2e-xp-rabbit-mab/4858
p-SLP76 pY128 J141-668.36.58 558367 BD https://www.bdbiosciences.com/en-us/products/reagents/western-blotting-and-molecular-reagents/western-blot-reagents/purified-mouse-anti-slp-76-py128.558367
p-Smad2 pS465/467 E8F3R 18338BF CST https://www.cellsignal.com/products/primary-antibodies/phospho-smad2-ser465-ser467-e8f3r-rabbit-mab/18338
p-STAT1 pY701 4a 612233 BD https://www.bdbiosciences.com/en-us/products/reagents/microscopy-imaging-reagents/immunofluorescence-reagents/purified-mouse-anti-stat1-py701.612233
p-STAT3 pY705 4/p-STAT3 612357 BD https://www.bdbiosciences.com/en-us/products/reagents/flow-cytometry-reagents/research-reagents/single-color-antibodies-ruo/purified-mouse-anti-stat3-py705.612357
p-STAT5 pY694 47/STAT5 611965 BD https://www.bdbiosciences.com/en-us/products/reagents/western-blotting-and-molecular-reagents/western-blot-reagents/purified-mouse-anti-human-stat5-py694.611965
p-ZAP70/p-SYK ZAP(pY139)/SYK(pY352) 17A 612575 BD https://www.bdbiosciences.com/en-us/products/reagents/flow-cytometry-reagents/research-reagents/single-color-antibodies-ruo/purified-mouse-anti-human-zap-70-py319-syk-py352.612575
pCD28 pY191 E5B9Z 16399BF CST https://www.cellsignal.com/products/primary-antibodies/phospho-cd28-tyr191-e5b9z-rabbit-mab/16399
PDGFR-β PR7212 MAB1263 R&D https://www.rndsystems.com/products/human-pdgf-rbeta-antibody-pr7212_mab1263?gad_source=1&gclid=Cj0KCQjwq86wBhDiARIsAJhuphk7_Od0oLnobNK4mzVXYXeOsNr1ADMBkgxjeEa-5hleSxcQ5iyqkqYaArgKEALw_wcB&gclsrc=aw.ds
renin 9 MA529523 ThermoFisher https://www.thermofisher.com/antibody/product/Renin-Antibody-clone-009-Recombinant-Monoclonal/MA5-29523
Smad2/3 D7G7 8685BF CST https://www.cellsignal.com/products/primary-antibodies/smad2-3-d7g7-xp-rabbit-mab/8685
Smad4 D3R4N 46535BF CST https://www.cellsignal.com/products/primary-antibodies/smad4-d3r4n-xp-rabbit-mab/46535
Snail/Slug poly ab180714 Abcam https://www.abcam.com/products/primary-antibodies/snail--slug-antibody-ab180714.html
uromodulin Polyclonal AF5144 R&D https://www.rndsystems.com/products/human-uromodulin-antibody_af5144
vimentin D21H3 5741BF CST https://www.cellsignal.com/products/primary-antibodies/vimentin-d21h3-xp-rabbit-mab/5741
Zeb1 3G6 14-9741-82 Thermofisher https://www.thermofisher.com/antibody/product/ZEB1-Antibody-clone-3G6-Monoclonal/14-9741-82
α-SMA 1A4 ab7817 Abcam https://www.abcam.com/products/primary-antibodies/alpha-smooth-muscle-actin-antibody-1a4-ab7817.html
β-catenin E-5 sc-133256 Santa Cruz https://www.scbt.com/p/occludin-antibody-e-5

Eukaryotic cell lines

Policy information about cell lines and Sex and Gender in Research

Cell line source(s): HEK293T cells and Jurkat cells were obtained from ATCC. Promega PowerPlex 18D System was used to confirm the identity of cell lines by STR (Short Tandem Repeat) analyses. Py2T cells were kindly provided as a gift from Dr. Christofori at the University of Basel, where this cell line was originally developed.

Authentication: ATCC thoroughly authenticates every cell line they provide. Py2T cells were developed and authenticated by Dr. Christofori.

Mycoplasma contamination: The cell lines were tested @ATCC for mycoplasma contamination and no mycoplasma was detected. In addition, the Tissue Culture Core at Wyss Institute performs regular mycoplasma tests and no cell line used in this study has been tested positive for mycoplasma.

Commonly misidentified lines (See ICLAC register): No commonly misidentified lines used in this study.

Flow Cytometry

Plots

Confirm that:

☒ The axis labels state the marker and fluorochrome used (e.g. CD4-FITC).

☒ The axis scales are clearly visible. Include numbers along axes only for bottom left plot of group (a 'group' is an analysis of identical markers).

☒ All plots are contour plots with outliers or pseudocolor plots.

☒ A numerical value for number of cells or percentage (with statistics) is provided.

Methodology

Sample preparation	All sample preparation steps were described in Methods.
Instrument	Data were acquired by a BD Fortessa flow cytometer.
Software	FACSDiva Software was used for flow cytometry data collection.
Cell population abundance	No sorting has been performed.
Gating strategy	Gating strategies for GFP positive and negative populations were described in Fig. S3. Gating strategies for dye dilution-based T cell proliferation assay were described in Fig. S8.

☒ Tick this box to confirm that a figure exemplifying the gating strategy is provided in the Supplementary Information.
