# Supplementary material for: Signal amplification by cyclic extension enables high-sensitivity single-cell mass cytometry
Source: Nat Biotechnol. Author manuscript; Available in PMC 2025 May 18. (PMC11910986; doi:10.1038/s41587-024-02316-x)
Supplement: Supplementary file 1 [file NIHMS2060292-supplement-Supplementary_Material.pdf]

nature biotechnology

Supplementary information https://doi.org/10.1038/s41587-024-02316-x

Signal amplification by cyclic extension enables high-sensitivity single-cell mass cytometry

In the format provided by the authors and unedited

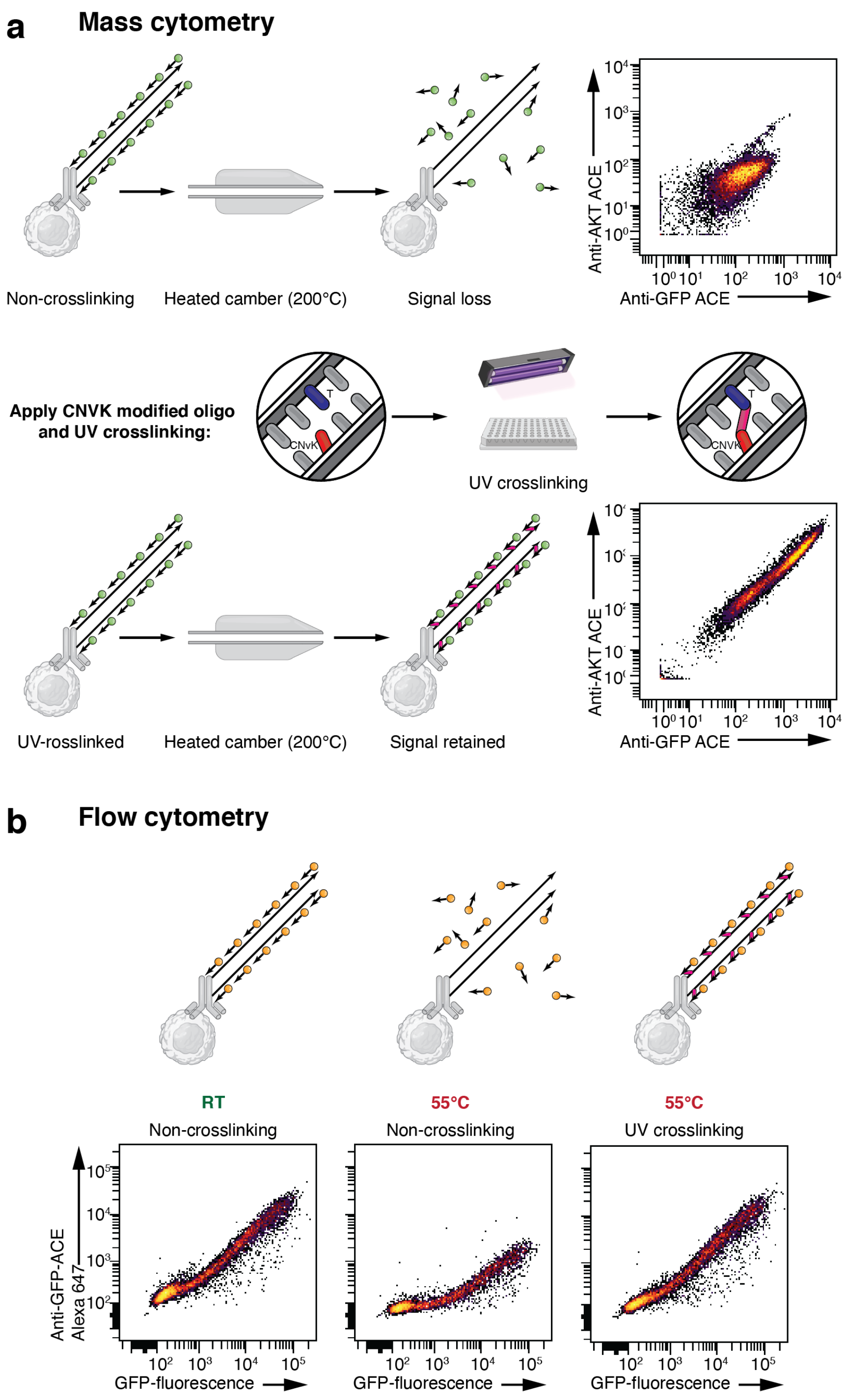

Fig. S1. a, ACE amplification DNA complexes were dissociated during the vaporization process in mass cytometry sample acquisition, resulted in low signal and high background. CNVK-based photo-crosslinking allows hybridized detectors to stay intact, which maintained the amplified signals. **b**, The same phenomenon was reproduced in flow cytometry using GFP-transfected cells. We observed that one-minute incubation at 55°C caused signal reduction of over 90%. By applying CNVK modification and UV crosslinking, this signal loss was rescued.

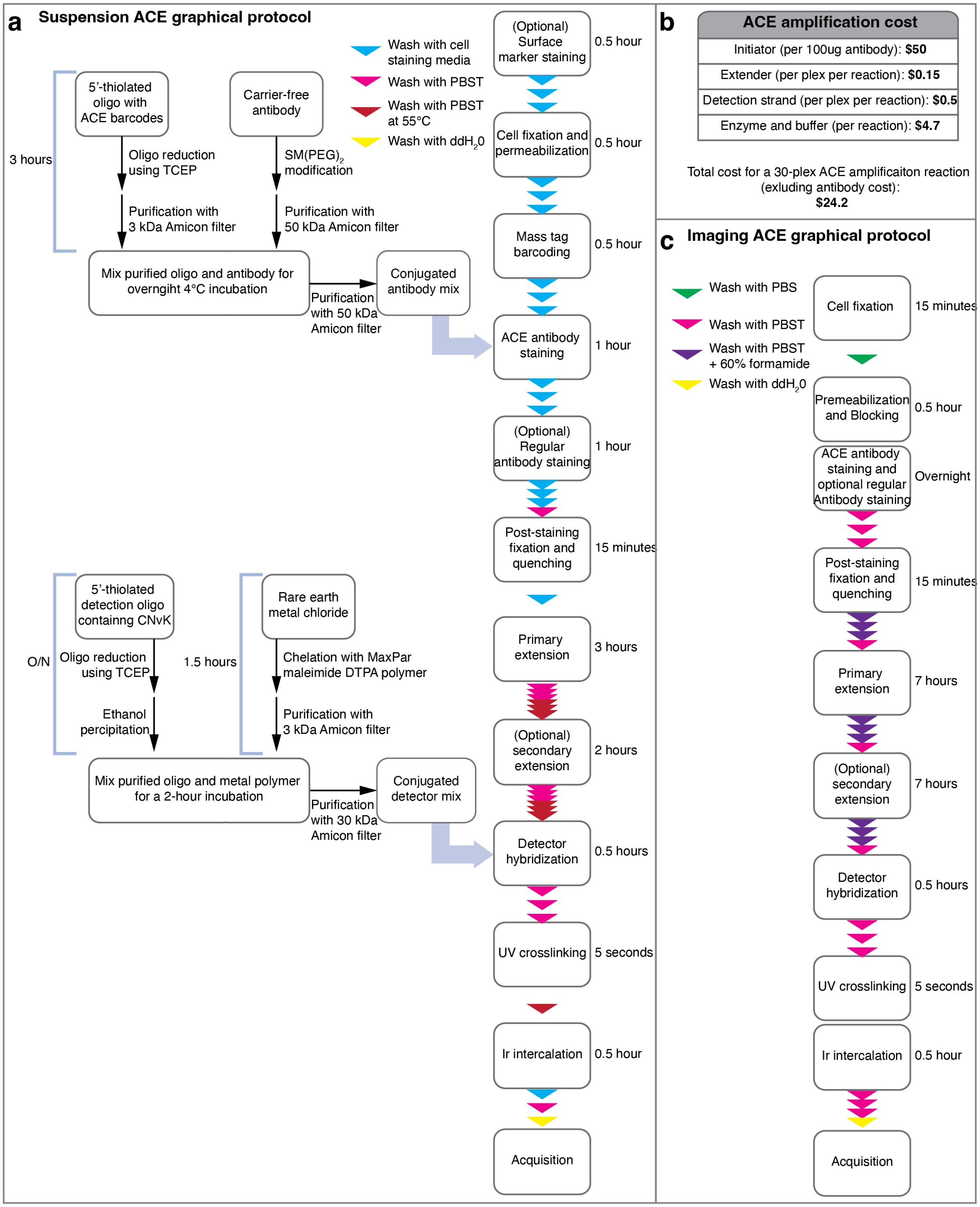
a
Suspension ACE graphical protocol
Wash with cell staining media
Wash with PBST
Wash with PBST at 55°C
Wash with ddH$_2$0
3 hours
5’-thiolated oligo with ACE barcodes
Oligo reduction using TCEP
Purification with 3 kDa Amicon filter
Carrier-free antibody
SM(PEG)$_2$ modification
Purification with 50 kDa Amicon filter
Mix purified oligo and antibody for overngiht 4°C incubation
Purification with 50 kDa Amicon filter
Conjugated antibody mix
O/N
5’-thiolated detection oligo containng CNvK
Oligo reduction using TCEP
Ethanol percipitation
1.5 hours
Rare earth metal chloride
Chelation with MaxPar maleimide DTPA polymer
Purification with 3 kDa Amicon filter
Mix purified oligo and metal polymer for a 2-hour incubation
Purification with 30 kDa Amicon filter
Conjugated detector mix
(Optional) Surface marker staining
0.5 hour
Cell fixation and permeabilization
0.5 hour
Mass tag barcoding
0.5 hour
ACE antibody staining
1 hour
(Optional) Regular antibody staining
1 hour
Post-staining fixation and quenching
15 minutes
Primary extension
3 hours
(Optional) secondary extension
2 hours
Detector hybridization
0.5 hours
UV crosslinking
5 seconds
Ir intercalation
0.5 hour
Acquisition
b
ACE amplification cost
Initiator (per 100ug antibody): **$50**
Extender (per plex per reaction): **$0.15**
Detection strand (per plex per reaction): **$0.5**
Enzyme and buffer (per reaction): **$4.7**
Total cost for a 30-plex ACE amplificaiton reaction (exluding antibody cost):
$24.2
c
Imaging ACE graphical protocol
Wash with PBS
Wash with PBST
Wash with PBST + 60% formamide
Wash with ddH$_2$0
Cell fixation
15 minutes
Premeabilization and Blocking
0.5 hour
ACE antibody staining and optional regular Antibody staining
Overnight
Post-staining fixation and quenching
15 minutes
Primary extension
7 hours
(Optional) secondary extension
7 hours
Detector hybridization
0.5 hours
UV crosslinking
5 seconds
Ir intercalation
0.5 hour
Acquisition

Fig. S2. Graphical schematics and the cost breakdown of ACE amplification. a, A graphical schematic of ACE signal amplification protocol for suspension mass cytometry, including the procedures of antibody conjugation, detection oligo conjugation, sample staining, and barcode oligo amplification. **b,** The cost breakdown of ACE reagents to stain one million cells in suspension or a tissue section sample on a slide. In total, a 30-plex ACE amplification reaction costs $24.2. **c,** A graphical schematic of ACE signal amplification protocol for imaging mass cytometry-based spatial profiling.

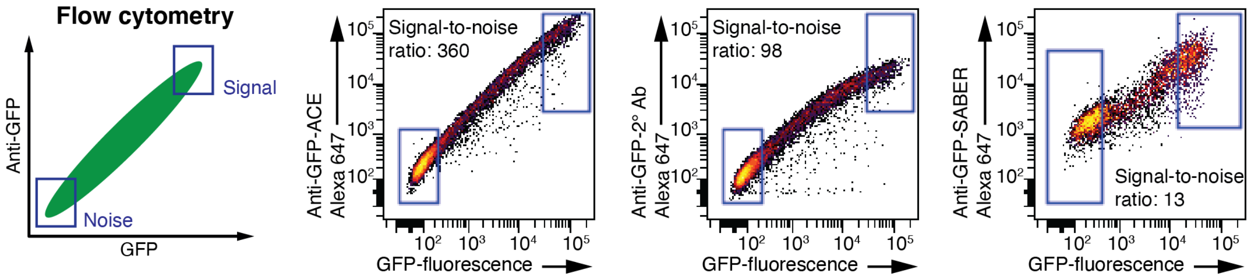

Fig. S3. a, GFP-transfected cells were analyzed using fluorescently tagged ACE detection oligos (anti-GFP-ACE), conventional fluorescent antibodies (anti-GFP secondary antibodies) or anti-GFP antibodies amplified using immuno-SABER to assess the signal-to-noise ratios. The performance of ACE was superior to secondary antibody amplification and immuno-SABER amplification.

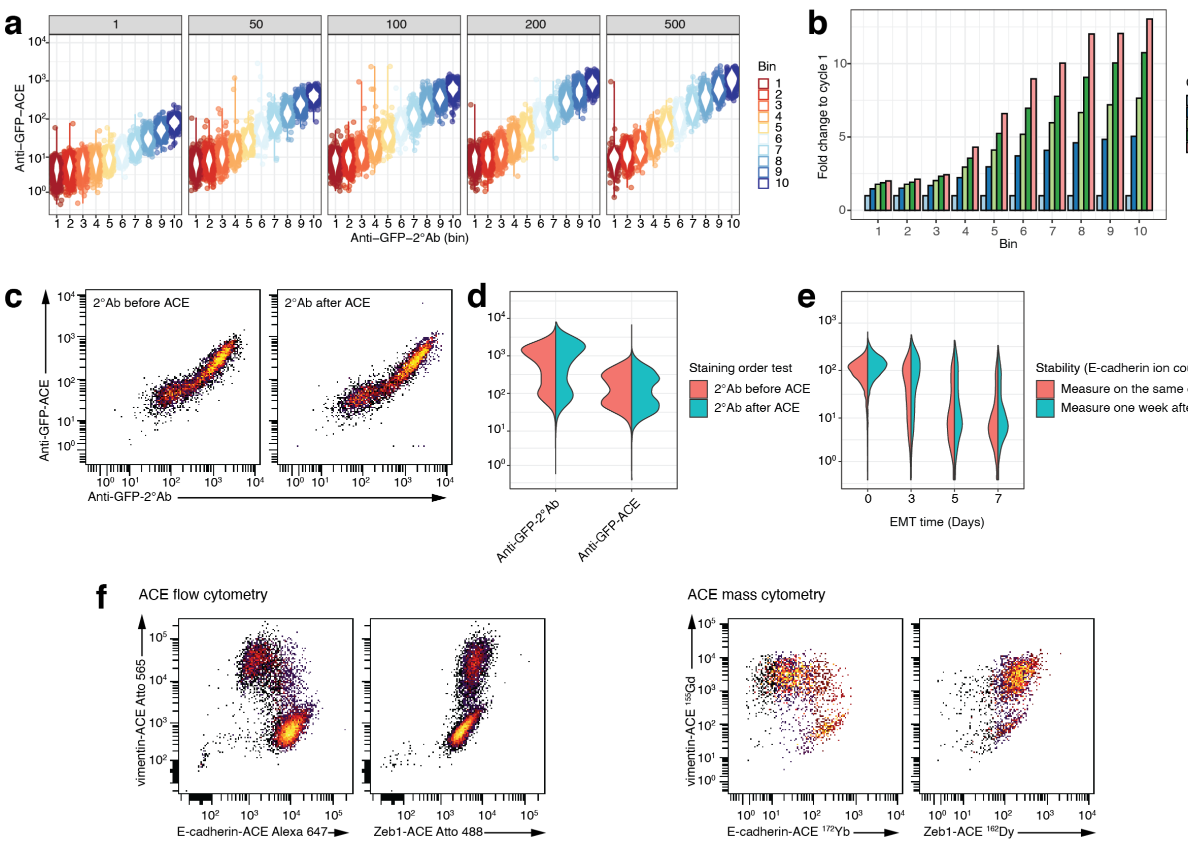

Fig. S4. Assessment of the ACE signal amplification method on mass cytometry. a, The 500 thermal-cycle time series data were divided into 10 equal-width bins according to the GFP expression levels indicated by the secondary antibody. **b**, Fold changes for each bin through the 1-500 cycles are plotted as a bar graph. **c-d**, To test the compatibility of using the ACE protocol and the conventional mass cytometry staining protocol on the same samples, we stained GFP-expressing cells with the ACE oligo-conjugated anti-GFP primary antibody. The conventionally labeled secondary antibody was applied to the cells either before or after a 500-cycle ACE amplification. The relationships between ACE signals and the signals from the conventional secondary antibody in both staining orders were shown as bi-axial plots in (**c**). Signals from the tested staining orders were directly compared in the split violin plot in (**d**). **e**, Py2T cells undergoing EMT were stained with the anti-E-cadherin ACE antibody. After ACE thermal cycles, samples were directly used for detector hybridization and mass cytometry acquisition or stored at 4 °C for one week before the follow-up steps. Signals from both tested were compared in the split violin plot. **f**, Py2T cells were stained with ACE antibodies targeting E-cadherin, vimentin, and Zeb-1. After ACE thermal cycles, fluorophore-labeled or metal labeled detections strands were hybridized to the cells before they were analyzed with a flow cytometer or a mass cytometer, respectively.

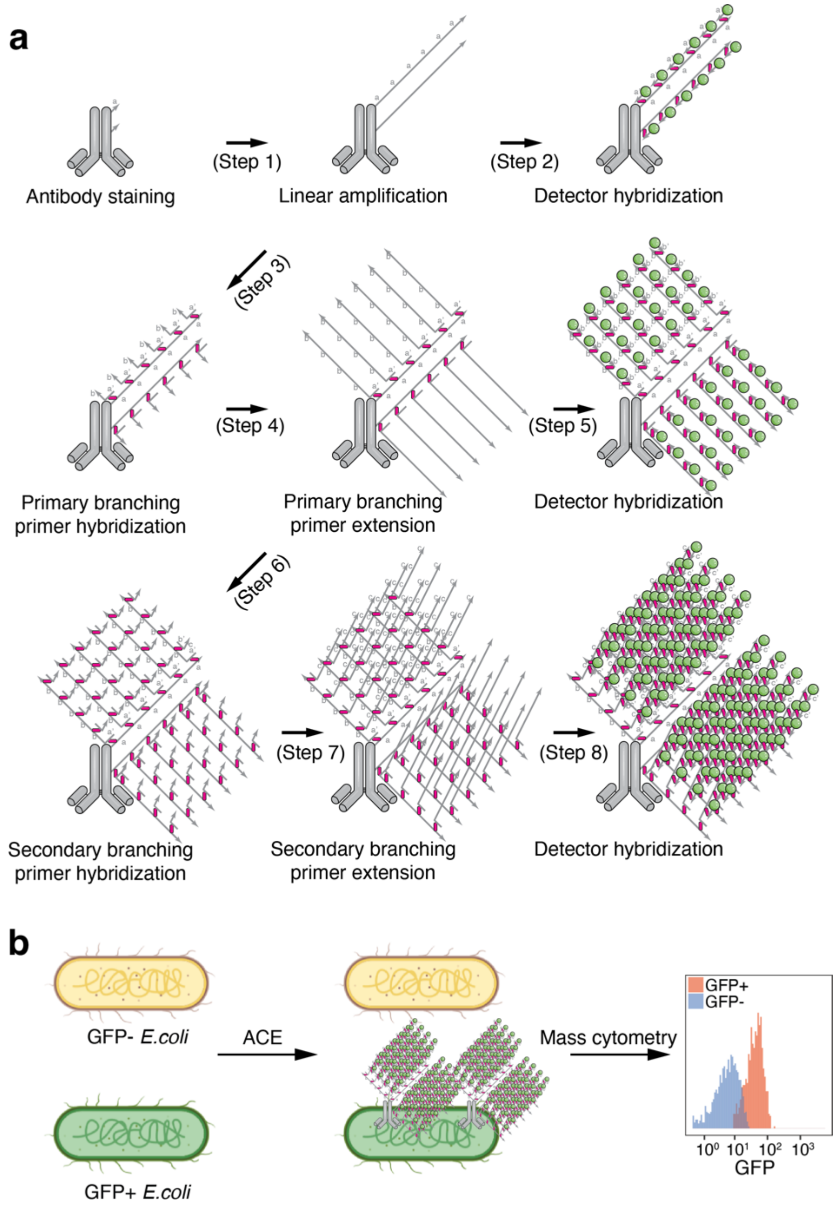

Fig. S5. Enhanced signal amplification by branching ACE amplification. a, After the linear extension (Step 1), detectors can be applied directly for mass cytometry analysis (Step 2). When branching is required to further amplify signals, the number of detector binding sites on each antibody can be expanded by applying the CNVK-containing primary branching primer (Step 3), followed by UV crosslinking and thermal cyclic extension (Step 4). Detectors can then be applied before mass cytometry analysis (Step 5). Based on the same principle, secondary branching (Step 6-8) can be implemented to ultimately generate over 500-fold signal enhancement in tested cells, compared to the unamplified signal. **b,** With two rounds of branching, protein abundances in microbes can be measured using mass cytometry as indicated by an experiment to analyze GFP expression levels in GFP+ and GFP- *E. coli* single cells.

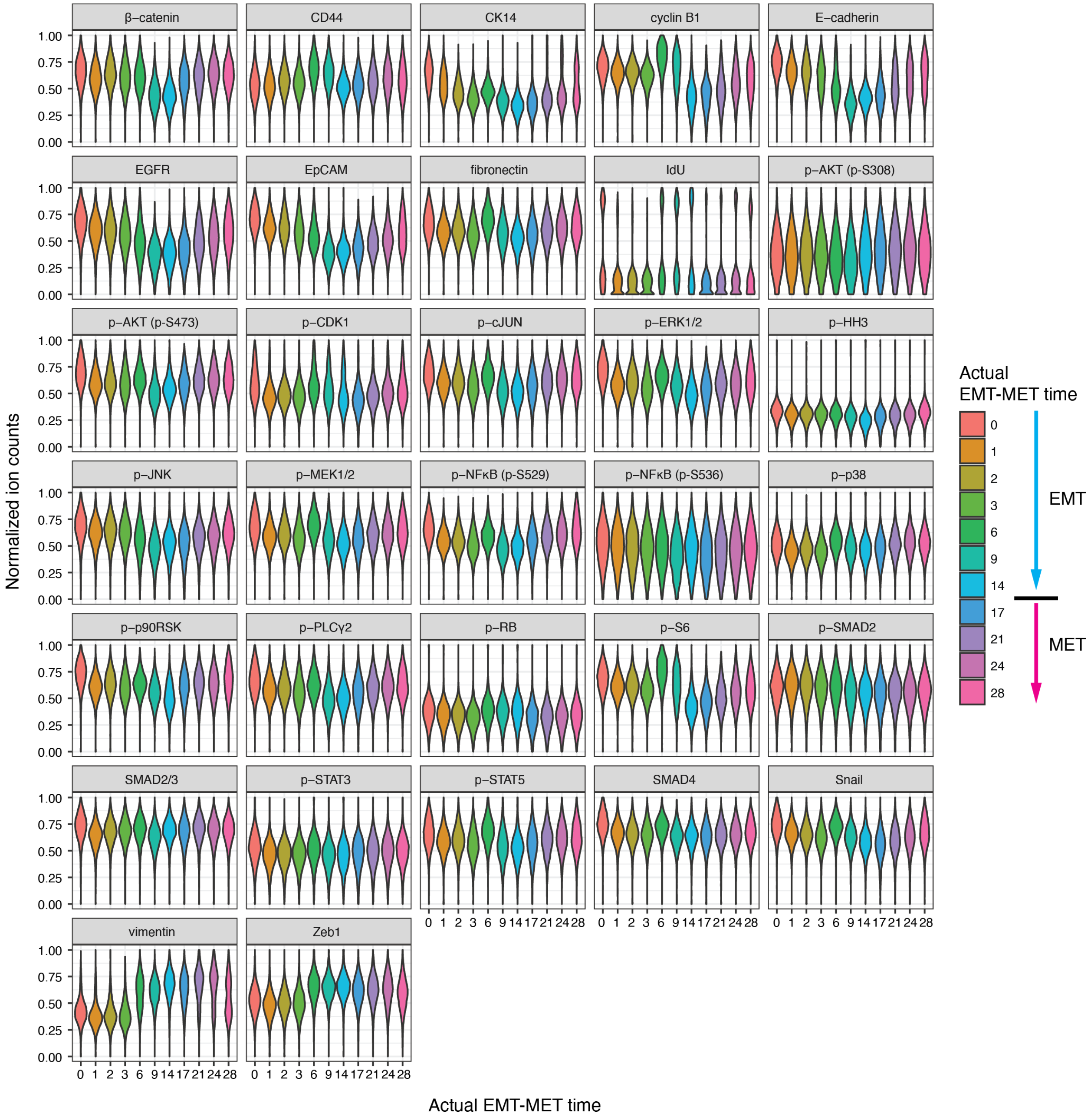

Fig. S6. ACE was performed on Py2T cells undergoing EMT and MET. The modulations of all 32 measured markers are shown as violin plots.

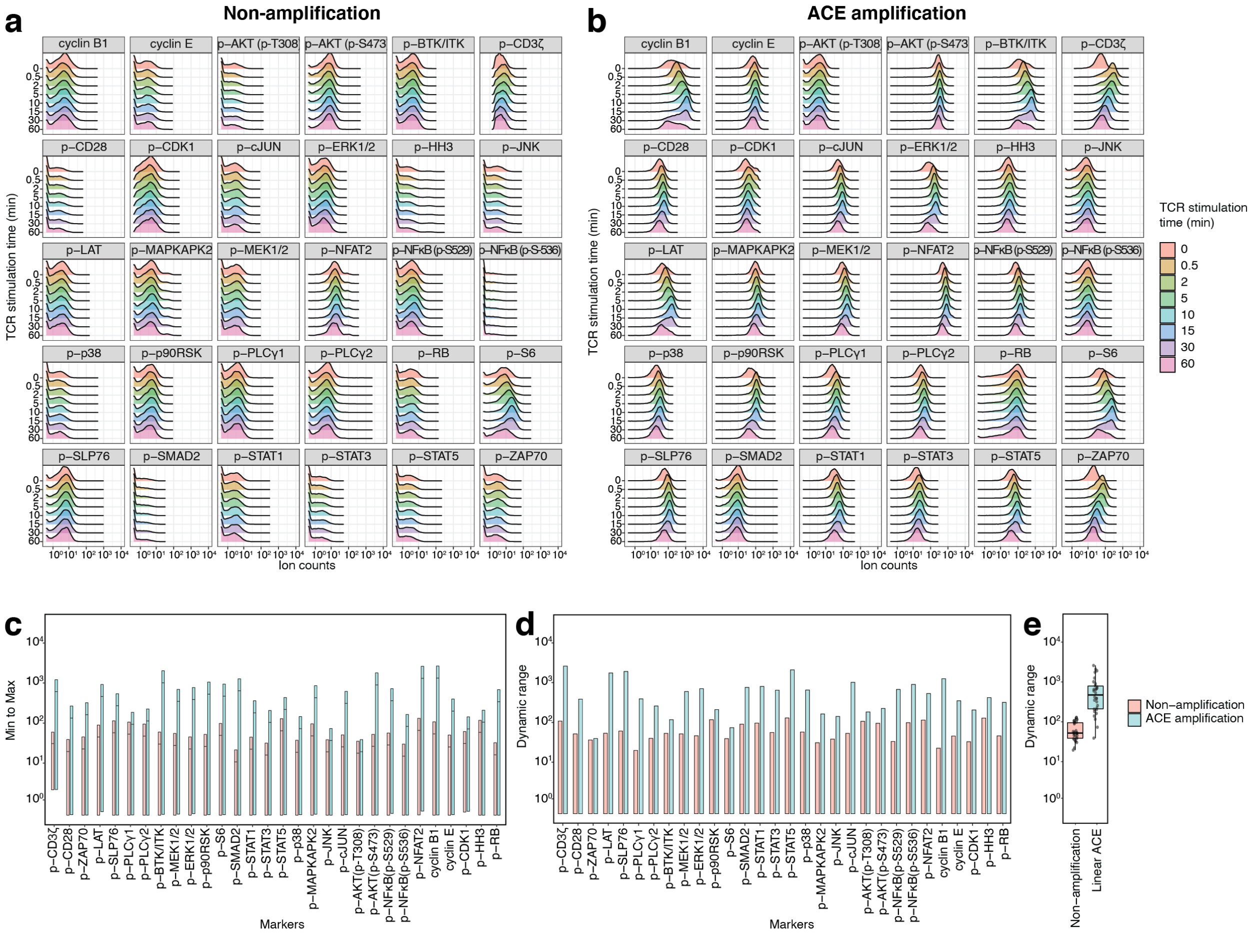

Fig. S7. ACE was used to enable multi-parametric signaling response analysis in Jurkat T cells. a-b, Signaling behaviors following TCR stimulation could be observed and quantified after ACE amplification (**b**). In comparison, only a few markers could be quantitatively analyzed in non-amplification samples (**a**). **c-e**, We quantified the dynamic range of each target of interest before and after ACE amplification, and the results were shown as (**c**) min and max signals, (**d**) dynamic ranges, and (**e**) averaged dynamic ranges across all channels with or without ACE amplification. The boxplot presents the first quartile (Q1), median, and third quartile (Q3). The inter-quartile range (IQR) define distance between Q1 and Q3. The upper whisker extends from the hinge to the largest value no further than 1.5*IQR from the hinge. The lower whisker extends from the hinge to the smallest value no further than 1.5*IQR of the hinge. All data points are overlaid onto the boxplot.

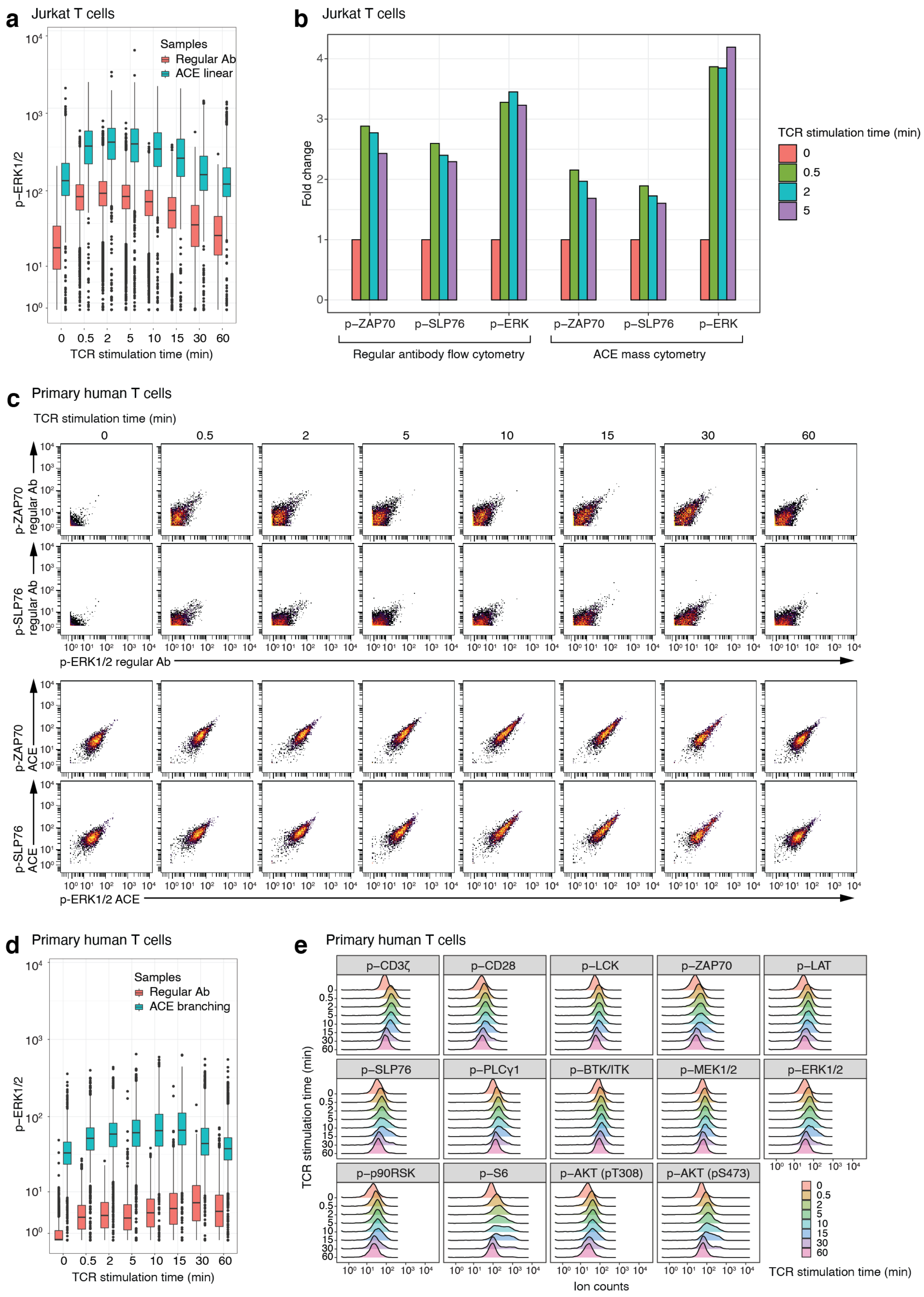

a
Jurkat T cells
Samples
Regular Ab
ACE linear
p–ERK1/2
TCR stimulation time (min)
b
Jurkat T cells
Fold change
TCR stimulation time (min)
p–ZAP70
p–SLP76
p–ERK
Regular antibody flow cytometry
ACE mass cytometry
c
Primary human T cells
TCR stimulation time (min)
p-ZAP70 regular Ab
p-SLP76 regular Ab
p-ERK1/2 regular Ab
p-ZAP70 ACE
p-SLP76 ACE
p-ERK1/2 ACE
d
Primary human T cells
Samples
Regular Ab
ACE branching
p–ERK1/2
TCR stimulation time (min)
e
Primary human T cells
p–CD3ζ
p–CD28
p–LCK
p–ZAP70
p–LAT
p–SLP76
p–PLCγ1
p–BTK/ITK
p–MEK1/2
p–ERK1/2
p–p90RSK
p–S6
p–AKT (pT308)
p–AKT (pS473)
TCR stimulation time (min)
Ion counts

Fig. S8. Comparison of the ACE approach with conventional mass or flow cytometry methods in profiling the TCR responses in Jurkat cells and primary human T cells. **a**, p-ERK1/2 levels in Jurkat T cells over the 1-hour TCR stimulation time course were assessed with ACE or conventionally labeled mass cytometry antibodies. ACE amplification resulted in 7-fold signal enhancement over conventional antibodies. **b**, Stimulated and unstimulated Jurkat cells were analyzed using mass cytometry after ACE amplification or using conventional flow cytometry with fluorophore-labeled antibodies. Levels of p-ERK1/2, p-ZAP70, p-SLP76 from stimulated cells versus unstimulated cells were calculated as fold changes for both methods. **c**, Phosphorylation levels of p-ERK1/2, p-ZAP70, p-SLP76 in primary human $CD4^+$ T cells were measured using conventionally labeled mass cytometry antibodies or using branching ACE over a 1-hour stimulation time course. **d**, In primary human $CD4^+$ T cells, the ACE-amplified p-ERK1/2 ion counts increased by 10 times compared to signals generated with a conventionally labeled antibody. **e**, Signaling behaviors in primary human $CD4^+$ T cells following TCR stimulation could be observed and quantified after branching ACE amplification. Boxplots in (**a**) and (**d**) present the first quartile (Q1), median, and third quartile (Q3). The inter-quartile range (IQR) define distance between Q1 and Q3. The upper whisker extends from the hinge to the largest value no further than 1.5*IQR from the hinge. The lower whisker extends from the hinge to the smallest value no further than 1.5*IQR of the hinge. Data beyond the end of the whiskers are plotted individually.

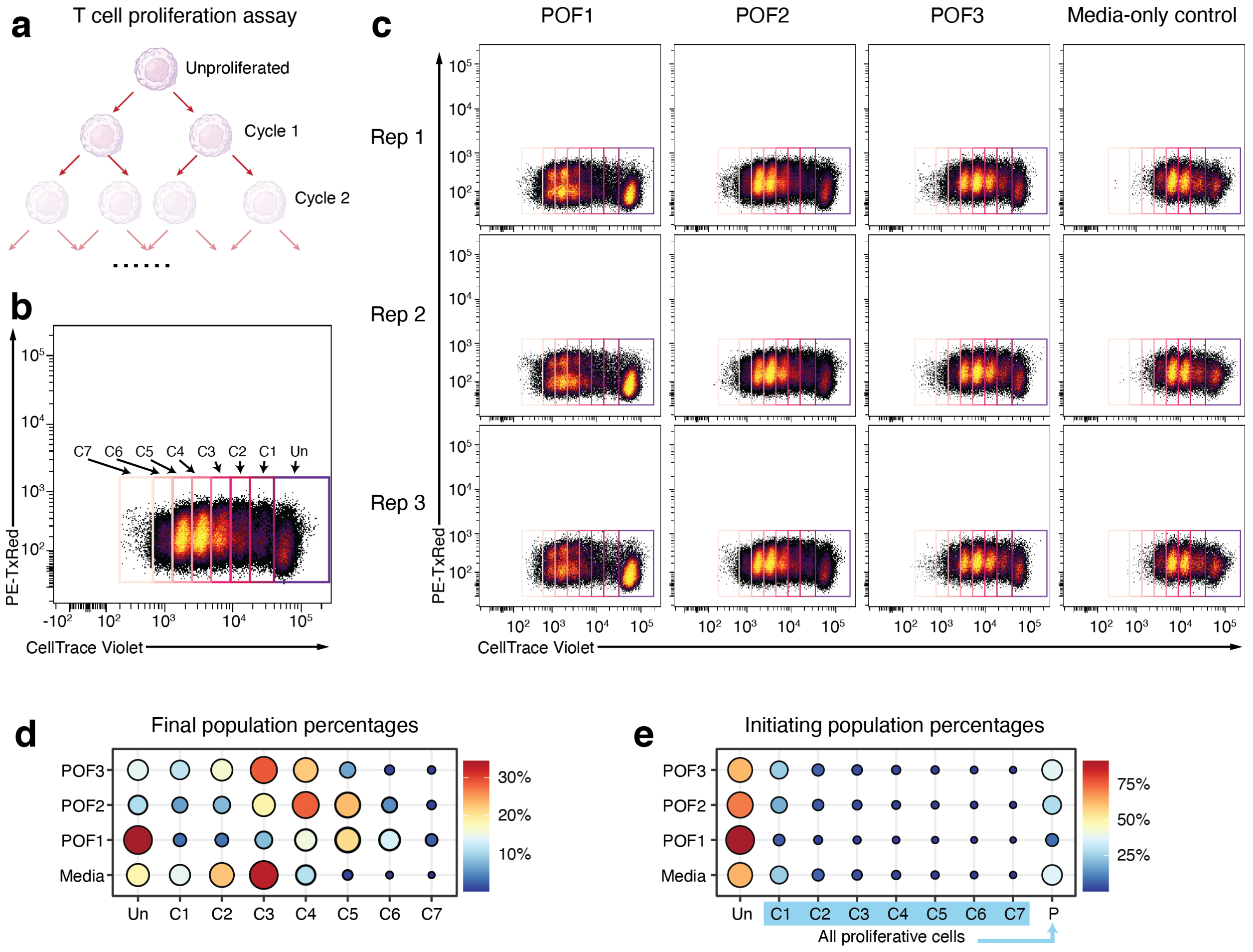

Fig. S9. Cell proliferation assay showed reduced proliferative capabilities in T lymphocytes cultured with patient POF samples. a, CellTrace Violet was used to stain isolated human T lymphocytes before they were activated with anti-CD3/anti-CD28 beads and cultured with POF samples for four days. T-cell proliferation was assessed by dye dilution on a flow cytometer to determine the number of generations through which a cell has progressed since the label was applied. **b**, An example gating strategy to show the generational populations of analyzed T cells. **c**, The same gating was applied on cells treated with all three individual POFs and a control media-only treatment condition across three replicates to determine (**d**) the percentages of the each generational population at the final state (i.e., the end point of the coculture experiment). **e**, Percentages of T cells at the initiating state (i.e., the beginning point of the coculture experiment) that are corresponding to determined generational populations were calculated to compute the percentages of proliferated T cells in response to the POF treatment.

a

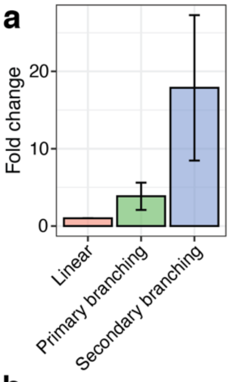

b

Antigen	Amplification methods
calbindin	L
nephrin	PB
EpCAM	L
p-S6	PB
α-SMA	L
vimentin	PB
uromodulin	L
COL1A1	L
fibronectin	L
agmatinase	L
CD31	PB
CD45	L
E-Cadherin	PB
ATP1A1	L
PDGFR-β	PB
claudin-1	L
nestin	PB
aquaporin1	PB
aquaporin2	PB
renin	PB

L: Linear
PB: Primary branching

c

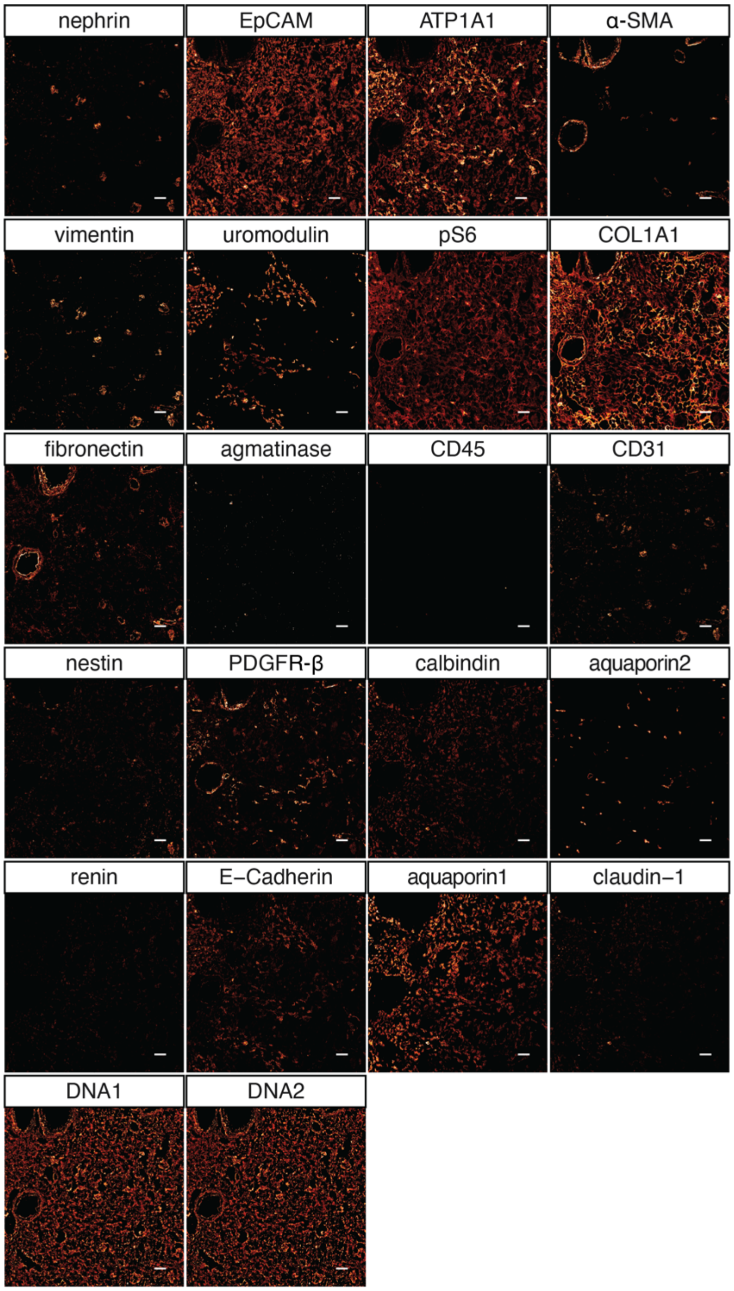

Fig. S10. ACE amplifies IMC signal for multi-parametric spatial profiling in cells and tissue samples. a, Benefits of additional branching amplifications for IMC are shown as signal fold changes to the linear ACE signal. Data are presented as mean values +/- SD. n = 5 individual cells. **b,** ACE was coupled with IMC to profile the human kidney tissue from a patient with renal cyst disease. Linear amplification or primary branching amplification were used on specific epitopes depending on their abundances in the sample. **c**, All analyzed markers are shown as images of individual channels. Experiments were performed in replicates to confirm the reproducibility. Scale bar, 200 µm.
